# Exofucosylation of Adipose Mesenchymal Stromal Cells Alters Their Secretome Profile

**DOI:** 10.3389/fcell.2020.584074

**Published:** 2020-11-26

**Authors:** David García-Bernal, Mariano García-Arranz, Ana I. García-Guillén, Ana M. García-Hernández, Miguel Blanquer, Damián García-Olmo, Robert Sackstein, Jose M. Moraleda, Agustín G. Zapata

**Affiliations:** ^1^Hematopoietic Transplant and Cellular Therapy Unit, Instituto Murciano de Investigación Biosanitaria Virgen de la Arrixaca, University of Murcia, Murcia, Spain; ^2^Department of Internal Medicine, Medicine School, University of Murcia, Murcia, Spain; ^3^Foundation Health Research Institute-Fundación Jiménez Díaz University Hospital, Madrid, Spain; ^4^Department of Translational Medicine, Herbert Wertheim College of Medicine, Florida International University, Miami, FL, United States; ^5^Department of Cell Biology, Faculty of Biology, Complutense University of Madrid, Madrid, Spain

**Keywords:** mesenchymal stromal cells, HCELL, adipose tissue, secretome, regenerative medicine

## Abstract

Mesenchymal stromal cells (MSCs) constitute the cell type more frequently used in many regenerative medicine approaches due to their exclusive immunomodulatory properties, and they have been reported to mediate profound immunomodulatory effects *in vivo*. Nevertheless, MSCs do not express essential adhesion molecules actively involved in cell migration, a phenotypic feature that hampers their ability to home inflamed tissues following intravenous administration. In this study, we investigated whether modification by fucosylation of murine AdMSCs (mAdMSCs) creates Hematopoietic Cell E-/L-selectin Ligand, the E-selectin-binding CD44 glycoform. This cell surface glycan modification of CD44 has previously shown in preclinical studies to favor trafficking of mAdMSCs to inflamed or injured peripheral tissues. We analyzed the impact that exofucosylation could have in other innate phenotypic and functional properties of MSCs. Compared to unmodified counterparts, fucosylated mAdMSCs demonstrated higher *in vitro* migration, an altered secretome pattern, including increased expression and secretion of anti-inflammatory molecules, and a higher capacity to inhibit mitogen-stimulated splenocyte proliferation under standard culture conditions. Together, these findings indicate that exofucosylation could represent a suitable cell engineering strategy, not only to facilitate the *in vivo* MSC colonization of damaged tissues after systemic administration, but also to convert MSCs in a more potent immunomodulatory/anti-inflammatory cell therapy-based product for the treatment of a variety of autoimmune, inflammatory, and degenerative diseases.

## Introduction

Mesenchymal stromal cells (MSCs) are a heterogeneous multipotent progenitor cell population found in almost all adult tissues throughout the body. MSCs possess the ability of self-renewal and mesodermal lineage differentiation (e.g., osteocytes, adipocytes, and chondrocytes), and mediate tissue repair through the production of different molecules with trophic actions (Caplan, 2007; Chamberlain et al., 2007). Remarkably, MSCs also exhibit unique anti-inflammatory and immunoregulatory properties by the secretion of non-conclusively determined anti-inflammatory molecules, including IL10, TGFβ, heme-oxygenase, IDO1, PGE_2_, HGF, galectin-1, and direct cell-to-cell contacts (Krampera et al., 2003; Yañez et al., 2010; Najar et al., 2016). Because of all these intrinsic features, MSCs have been considered a promising cell therapy product for a variety of systemic inflammatory disorders, including rheumatoid arthritis, graft-versus-host disease, multiple sclerosis, or type 1 diabetes (Lee et al., 2006; Yañez et al., 2006; Augello et al., 2007; Gerdoni et al., 2007; Abdi et al., 2015).

An important issue for adoptive cellular therapeutics is that the MSC immunomodulatory effectiveness is closely related to the ability of the systemically administered cells to traffic and infiltrate the inflamed tissues (Lee et al., 2006; Abdi et al., 2015). In mammals, microvascular endothelial beds at injured or inflamed tissues express E-selectin, whose expression is up-regulated by pro-inflammatory cytokines such as IL-1 and TNFα (Sackstein, 2009; Thankamony and Sackstein, 2011). E-selectin is a Ca^2+^-dependent endothelial lectin belonging to “selectins” family that prototypically bind sialofucosylated glycan determinants expressed on their respective ligands (i.e., glycoproteins and/or glycolipids), specifically the terminal tetrasaccharide motif sialyl Lewis X (sLe^*x*^). However, MSCs lack expression of ligands for E-selectin, such as sialofucosylated PSGL-1, CD43, and CD44 glycoforms, molecules called cutaneous lymphocyte antigen (CLA), CD43E, and Hematopoietic Cell E-/L-selectin Ligand (HCELL), respectively, Sackstein et al. (2008). The absence of these molecular effectors of cell migration hinders the capacity of infused MSCs to extravasate into the inflamed tissues following systemic administration (Kang et al., 2012; Eggenhofer et al., 2014; Abdi et al., 2015), limiting the efficacy of this immunomodulatory cell therapy. Therefore, it is important to design new cell engineering based-strategies for improving the tissue delivery of MSCs to inflamed tissues.

Mammalian MSCs characteristically express CD44, and stereospecific CD44 glycoengineering via fucosyltransferase-mediated fucose modification of N-acetylglucosamine results in the transient generation of the tetrasaccharide sLe^*x*^ (Sackstein et al., 2008; Abdi et al., 2015; Lopez-Lucas et al., 2018). Exofucosylation of CD44 on MSCs following treatment with either stereospecific fucosyltransferase-VI (FTVI) or -VII (FTVII) thereby creates the potent E-selectin ligand HCELL on MSC surface. This surface glycan modification of MSCs by exofucosylation has been previously shown to enhance tethering and rolling on E-selectin-bearing endothelial cells, and yields markedly increased *in vivo* murine and human MSC homing to endothelial beds that express E-selectin such as bone marrow and inflamed/injured tissues (Sackstein et al., 2008; Abdi et al., 2015; Chou et al., 2017).

However, to date, apart from its augmented E-selectin binding ability and the consequent improved *in vivo* migration capacity to inflamed tissues, other phenotypic and functional properties of MSCs after exofucosylation have not been tested. To address this question, in the present work we performed a direct comparison between unmodified murine AdMSCs (UmAdMSCs, i.e., HCELL^–^ mAdMSCs) and fucosylated mAdMSCs (FucmAdMSCs, i.e., HCELL^+^ mAdMSCs), by analyzing their cell morphology in culture, proliferation kinetics, MSC immunophenotype, multipotent differentiation capacity, expression of anti-inflammatory factors, secretory profiles, anti-proliferative effect on mitogen-stimulated splenocytes, wound healing effects and migration *in vitro*.

## Materials and Methods

### Animals

C57BL/6 and β-actin-GFP transgenic C57BL/6-Tg(CAG-EGFP) mice were purchased from Envigo (Huntingdon, United Kingdom) and Jackson Laboratory (Bar Harbor, ME, United States), respectively, and housed in a pathogen-free environment at the animal supply facilities of the University of Murcia and Foundation Health Research Institute-Fundación Jiménez Díaz University Hospital.

### Isolation of Adipose Murine Mesenchymal Stromal Cell and Culture

Murine adipose tissue-derived mesenchymal stromal cells (mAdMSCs) were isolated as reported previously (Yañez et al., 2006). Briefly, adipose tissue was excised from the epididymal fat pads, cut into small fragments, digested with 0.1% collagenase-A (Roche Diagnostics, Basel, Switzerland) in serum-free DMEM for 2 h at 37°C, filtered through 40-μm nylon cell strainers and cultured in MesenCult, a specific medium for the culture of mouse MSCs (Stem Cell Technologies, Vancouver, Canada), containing 1% L-glutamine (Gibco, Carlsbad, CA, United States), 100 U/ml penicillin, 100 μg/ml streptomycin (Gibco), 10% serum, and growth factor supplement (Stem Cells Technologies; complete medium). For experiments using IFNγ-stimulated mAdMSCs, cells were incubated with 500 U/ml recombinant murine IFNγ (R&D Systems, Minneapolis, MN, United States) for 24 h at 37°C.

### Murine AdMSC Exofucosylation

Murine AdMSCs were resuspended at 2 × 10^7^ cells/ml in fucosyltransferase VII (FTVII) reaction buffer composed of Hanks Balanced Salt Solution (HBSS; without Ca^2+^/Mg^2+;^ Lonza, Basel, Switzerland) containing 30 μg/ml FTVII (R&D Systems), 20 mM HEPES (Sigma Aldrich, St. Louis, MO, United States), 0.1% human serum albumin (Sigma Aldrich), and 1 mM GDP-fucose (Sigma Aldrich), and treated for 60 min at 37°C (FucmAdMSCs). Negative controls were mAdMSCs treated only with reaction buffer (w/o FTVII and GDP-fucose, i.e., UmAdMSCs), mAdMSCs treated only with GDP-fucose (w/o FTVII, i.e, GDP-fuc-mAdMSCs) and mAdMSCs treated only with FTVII (w/o GDP-fucose, i.e., FTVII-mAdMSCs). Concentrations of FTVII and GDP-fucose to achieve an optimal efficiency of the exofucosylation protocol were determined (Supplementary Figure 1).

Cell viability after fucosylation was assessed by trypan blue exclusion and 7-AAD staining (usually ≥ 95% live cells). Efficacy of FTVII modification was evaluated by analysis of HECA452 antibody (BD Biosciences, San Jose, CA, United States) reactivity and Ca^2+–^dependent mE-IgG chimera (R&D Systems) binding by flow cytometry. In some experiments, mAdMSCs after fucosylation were digested with 0.1 U/ml *Vibrio cholerae* sialidase (Roche Diagnostics) for 1 h at 37°C to remove terminal sLe^*x*^ decorations from cell surface glycoproteins, or with the broad-spectrum peptidases bromelain (Sigma-Aldrich) and proteinase K (Roche Diagnostics) to analyze whether E-selectin binding is mediated by glycoproteins and/or glycolipids.

### Western Blot Analysis

UmAdMSCs and FucmAdMSCs lysates were prepared with lysis buffer containing 150 mM NaCl, 50 mM Tris–HCl (pH 7.4), 0.2% SDS, 2% NP-40 and protease inhibitor cocktail (Thermo Scientific, Waltham, MA, United States). The cell lysates were resolved on 4–20% precast polyacrylamide gels (Bio-Rad, Hercules, CA, United States) and transferred onto PVDF blotting membranes (GE Healthcare Life Science, Barcelona, Spain). Then, membranes were blocked in TBS containing 0.1% Tween-20 (Sigma Aldrich) and 5% skim milk powder, incubated with mE-IgG chimera or anti-mouse CD44 primary antibody (clone IM7, Thermo Scientific) and then with HRP-conjugated secondary antibodies. Finally, protein bands were detected by chemiluminiscence using Lumi-Light Western Blotting Substrate (Roche Diagnostics) according to the manufacturer’s instructions.

### Analysis of Secretome and ELISAs Assays

For secretome analysis, UmAdMSCs or FucmAdMSCs were plated at a cell density of 1 × 10^4^ cells/cm^2^ and incubated in complete medium at 37°C. When cultures were 70–80% confluent, cells were washed three times with PBS and re-fed with serum-free media. After, conditioned media were collected at different times of culture (from 0 h up to 72 h), centrifuged at 200 *g* for 5 min, filtered through a 0.22-μm syringe filter and stored at -80°C until be analyzed. Finally, analysis of the secretome of UmAdMSCs or FucmAdMSCs was performed using the Bio-Plex Pro mouse cytokine, chemokine, and growth factor magnetic bead-based assays (Bio-Rad) in a Luminex system (Milliplex MAP, Merck, Darmstadt, Germany), according to manufacturer instructions. Raw data of each analyte concentration are shown in Supplementary Table 1. To evaluate the ability of UmAdMSCs and FucmAdMSCs to secrete anti-inflammatory molecules by ELISA assays, supernatants were obtained culturing cells in the same conditions as above in complete medium for 72 h at 37°C. Levels of TGFβ, IL10, PGE_2_, IDO1, galectin-1, HGF, and heme-oxygenase (HO) were measured in the supernatants using specific mouse ELISA kits purchased from Abcam (Cambridge, United Kingdom), Cusabio Biotech (Houston, TX, United States), Diaclone (Bionova Cientifica, Madrid, Spain), and Elabscience (Bethesda, MD, United States).

### Quantitative Real-Time PCR

Complementary DNA (cDNA) from UmAdMSCs and FucmAdMSCs cultured in complete medium for 24 h was synthesized from total mRNA, and gene expression was analyzed by qRT-PCR using SYBR Green reagent (Takara, Kusatsu, Japan) in an iQ5 real-time PCR detection system (Bio-Rad). PCR thermal cycler conditions included a hold step of initial denaturation at 95°C for 10 s followed by two-step protocol consisting of 40 cycles of denaturation at 95°C for 5 s and annealing/extension at 60°C for 20 s. To control differences on input mRNA amounts, the mouse Gapdh was used as housekeeping gene to quantify and normalize the results. Fold-change gene expression was assessed by the 2^–Δ^ ^Δ^ ^*Ct*^ method. The primer sequences used are listed in Supplementary Table 2.

### Mitogen Stimulated Splenocyte Proliferation Assays

Splenocytes were isolated by mechanical dissociation of the spleen of C57BL/6 mice, followed by passage through 40-μm cell strainers (BD Biosciences) and red blood cell lysis with 0.8% ammonium chloride solution (Sigma Aldrich). Then, splenocytes were resuspended in RPMI 1640 medium (Lonza) containing 10% fetal bovine serum (FBS; Lonza), plated at 1 × 10^5^ cells/well in 96-well plates and stimulated to proliferate with 10 μg/ml concanavalin A (ConA; Sigma Aldrich). To evaluate the immunosuppressive properties of mAdMSCs on mitogen-induced splenocyte proliferation, five MSC:splenocyte increasing ratios were used: 1:100, 1:50, 1:25, 1:10, and 1:1. mAdMSC were previously irradiated at 15 Gy to avoid their proliferation while preserving their viability. 3 days after co-cultures, splenocyte proliferation was quantified using an ELISA BrdU colorimetric kit (Roche Diagnostics), according to the manufacturer’s instructions.

### Proliferation Assays (MTT)

The proliferation of UmAdMSCs or FucmAdMSCs was evaluated using the standard colorimetric MTT assays up to 7 days of cell culture. In brief, cells were seeded at 1.5 × 10^3^ cells/well in 96-well plates and allowed to attach for 24 h. To determine cell proliferation, wells were rinsed twice with PBS and incubated with MTT reagent (5 mg/ml; Sigma Aldrich) resuspended in DMEM w/o phenol red (Sigma Aldrich) for 4 h at 37°C. Then, 100 μl DMSO (Sigma Aldrich) was used to solubilize the formazan. Finally, absorbance at 565 nm was measured. Each condition was analyzed in quadruplicate. Alternatively, proliferation kinetics of both types of mAdMSCs was analyzed by calculating the cumulative population doubling (PD) level using the formula: $\text{PD}=\left(\frac{1}{\log_{10}2}\right)\times \log_{10}\left(\frac{\text{Nt}}{\text{No}}\right)$ where *No* represents the number of viable cells at seeding and *Nt* the number of viable cells at harvest, as previously reported (Christodoulou et al., 2013).

### Mesenchymal Stromal Cell Immunophenotyping

Murine AdMSCs were characterized by flow cytometry. Three different pools of mAdMSCs (each of them isolated from 10 to 15 mice) and at different culture passages (from passage 2 up to passage 5) were analyzed the same day, and at least 5 × 10^4^ events for all samples were recorded. Briefly, cells were detached with TrypLE Express dissociation reagent (Gibco), washed, and resuspended in PBS (Gibco) containing 1% fetal bovine serum (Lonza). Then, mAdMSCs were incubated with fluorochrome-conjugated anti-mouse antibodies specific for the surface markers CD73, CD90, CD105, CD44, PSGL-1, CD43, CD29, Sca-1, CD106, CD166, CD34, CD45, CXCR4, c-Kit, CD80, CD86 (all from BioLegend, San Diego, CA, United States) and for the tetrasaccharide sialyl Lewis X/A (clone HECA452; BD Biosciences), or their specific control isotype antibodies, for 30 min in the dark at 4°C. Finally, cells were washed, resuspended at 1 × 10^6^ cells/ml and analyzed in a FACSCanto II flow cytometer (BD Biosciences).

### Multipotent Differentiation Assays

Murine AdMSCs were analyzed for their multipotent differentiation capacity into the adipogenic, osteogenic, and chondrogenic mesodermal lineages using the mouse mesenchymal stem cell functional identification kit (R&D Systems) following the manufacturer’s instructions. To determine whether UmAdMSCs and FucmAdMSCs differentiate into adipocytes, cells were cultured in MesenCult complete medium with adipogenic supplement containing hydrocortisone, isobutylmethylxanthine and indomethacin. After 14 days of culture, cells were fixed with 4% paraformaldehyde in PBS and stained with Oil Red O solution (Sigma Aldrich) to detect lipid-laden vacuoles. For osteogenic differentiation, cells were cultured in MesenCult complete medium with osteogenic supplement containing ascorbate-phosphate, β-glycerophosphate and recombinant human BMP-2. After 21 days of culture, cells were fixed with 4% paraformaldehyde in PBS and stained with Alizarin Red (Sigma Aldrich) to detect calcified matrix culture depositions, or with SigmaFAST^TM^ BCIP-NBT (Sigma Aldrich) to analyze alkaline phosphatase activity, respectively. For chondrogenic differentiation, mAdMSCs were cultured in serum-free DMEM-F12 medium (Gibco) with chondrogenic supplement containing dexamethasone, ascorbate-phosphate, proline, pyruvate, recombinant human TGF-β3, and 1% ITS supplement (insulin, transferrin, selenious acid, bovine serum albumin, and linoleic acid; R&D Systems). In brief, 1 × 10^6^ mAdMSCs were seeded in a 15-ml polypropylene tube and centrifuged to obtain a pellet. The pellets were cultured in chondrogenic medium or complete medium as negative control, changing medium every 2–3 days. After 21 days of culture, the pellets were fixed with 4% paraformaldehyde in PBS, dehydrated, embedded in paraffin and 5-μm-thick sections were cut. Chondrogenic differentiation was analyzed by Alcian blue staining (Sigma Aldrich) to detect acidic mucopolysaccharides.

These differentiation capacities were also demonstrated by qPCR in order to detect the expression of specific adipogenic, osteogenic, and chondrogenic genes in each mAdMSC sample.

### Scratch-Culture and Transwell Migration Assays

Scratch-culture migration assays were performed as previously described (Collado-Gonzalez et al., 2017). In brief, UmAdMSCs, GDP-fuc-mAdMSCs, FTVII-mAdMSCs, or FucmAdMSCs were seeded into 12-well plates at a density of 1.5 × 10^5^ cells/well and cultured for 12 h to obtain confluent cell monolayers. Thereafter, a scratch (wound) was performed in the confluent cell monolayer with a sterile 10-μl pipette tip, and rinsed three times with PBS to remove detached cells after wounding. The scratched cell monolayers were maintained in complete medium at 37°C. To measure the extent of cell migration, images of the wound area were captured at different times (i.e., 0, 24, and 48 h) with an inverted phase-contrast microscope (Nikon, Tokyo, Japan). ImageJ software (National Institutes of Health, Bethesda, MD, United States) was used to measure the wound area reduction after 24 h or 48 h related to the same wound area at 0 h.

Moreover, migration assays were carried out using the transwell system (8-μm pore polycarbonate membrane, NUNC, Roskilde, Denmark). The lower chamber contained MesenCult medium containing 0.3% bovine serum albumin (Sigma Aldrich) or 30% FBS as a positive control of cell migration. In these experiments, mAdMSCs were previously irradiated to inhibit their proliferation in presence of FBS and just analyze differences in cell migration. Also, the chemotactic activity of murine pro-inflammatory chemokines CCL5 (RANTES; 150 ng/ml), CCL11 (eotaxin; 100 ng/ml), CCL20 (MIP3α; 100 ng/ml), CCL22 (macrophage-derived chemokine, MDC; 100 ng/ml), and CXCL16 (50 ng/ml) on mAdMSCs migration was evaluated. Concentrations of chemokines in the lower chamber were chosen according to the optimal mAdMSC migration index showed in previous experiments. All murine recombinant chemokines was purchased from Novus Biologicals (Littleton, CO, United States). Briefly, 1 × 10^5^ UmAdMSCs, GDP-fuc-mAdMSCs, FTVII-mAdMSCs, or FucmAdMSCs were resuspended in MesenCult with 0.3% BSA and added to the upper chamber, followed by incubation at 37°C for 6 h to assess chemokine activity or for 24 h to measure spontaneous cell migration (BSA). Finally, extent of cell migration to the lower chamber was quantified using an automated cell counter (Bio-Rad TC20). Each condition was analyzed in triplicate. Data were represented as percentages of migrated cells related to the control (100% of migration).

### Statistical Analysis

Data from the different experiments are represented as mean ± standard deviation (SD). Comparisons of results between different experimental groups were analyzed using one-way ANOVA and Tukey’s *post-hoc* comparison tests. *P* values < 0.05 were considered significant.

## Results

### FTVII-Treatment of mAdMSCs Enforces HCELL Expression

**FIGURE 1:**
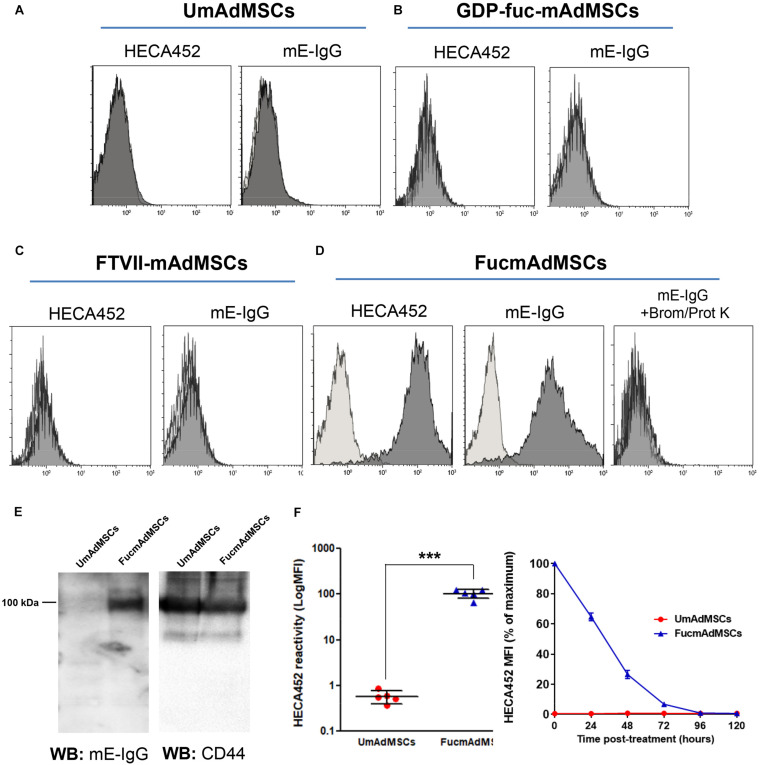
Exofucosylation induced E-selectin ligand expression in mAdMSCs. mAdMSCs were exofucosylated and analyzed for expression of sLe^*x*^ with HECA452 antibody and for Ca^2+^-dependent binding of the mE-IgG chimeric fusion protein, a reporter for E-selectin binding function. **(A)** Untreated mAdMSCs, **(B,C)** or only treated with GDP-fucose or FTVII, respectively, did not stain with HECA452 nor bound to mE-IgG, whereas FTVII and GDP-fucose treatment induced a substantial up-regulation of sLex expression (**D**, left) and a comparable binding of mE-IgG on mAdMSC surface (**D**, middle). Digestion of FucmAdMSCs with bromelain (Brom) and proteinase K (Prot K) significantly inhibits Ca^2+^-dependent mE-Ig binding (**D**, right). Control isotype antibody staining or mE-IgG binding with control buffer (w/o Ca^2+^) are represented as light gray histograms. **(E)** Western blot analysis of UmAdMSCs and FucmAdMSCs lysates to analyze mE-IgG reactivity (left). After, membranes were reproved against murine CD44 as protein loading control (right). (**F**, left) Different pools of mAdMSCs (*n* = 5) were fucosylated (FucmAdMSCs) or treated with buffer alone (UmAdMSCs) and analyzed for sLe^*x*^ expression with the HECA452 antibody by flow cytometry. Expression of sLe^*x*^ was significantly up-regulated, ***p < 0.001. (**F**, right) Kinetics of sLe^*x*^ surface expression after FTVII-treatment of mAdMSCs. UmAdMSCs or FucmAdMSCs were analyzed for sLe^*x*^ expression with the HECA452 antibody by flow cytometry up to 120 h post-fucosylation. MFI: Mean fluorescence intensity. Results are representative of three independent experiments and were analyzed using one-way ANOVA.

Flow cytometry analysis showed that unmodified mAdMSCs (i.e., UmAdMSCs) did not display any reactivity with the specific anti-sLe^*x*^ monoclonal antibody (mAb) HECA452. In addition, they did not bind to mouse E-selectin-IgG chimeric molecules (mE-IgG; Figure 1A). Negative controls including mAdMSCs treated with GDP-fucose alone (i.e., GDP-fuc-mAdMSCs, Figure 1B) and mAdMSCs treated with FTVII alone (i.e., FTVII-mAdMSCs, Figure 1C) neither showed HECA452 nor mE-IgG binding. However, exofucosylation enzymatic reaction provoked a substantial up-regulation of sLe^*x*^ expression on mAdMSCs (i.e., FucmAdMSCs), evidenced by an intense staining with the mAb HECA452 (Figure 1D, left), as well as a proportional Ca^2+^-dependent mE-IgG binding (Figure 1D, middle). Digestion of FucmAdMSCs with broad-spectrum peptidases [i.e., bromelain (Brom) and proteinase K (Prot K)] significantly inhibits mE-Ig binding, showing that glycoproteins are the main mE-IgG ligands rather than sLe^*x*^-decorated glycolipids (Figure 1D, right). Additional western blot analysis of whole FucmAdMSC lysates revealed that the main glycoprotein recognized by mE-IgG is the ∼100 kDa standard isoform of CD44 (i.e., CD44s; Figure 1E), as previously reported for human and mouse bone marrow MSCs (Sackstein et al., 2008; Abdi et al., 2015). Thus, exofucosylation of mAdMSCs induced expression of HCELL.

In order to evaluate the variability on CD44 conversion to HCELL glycoform, five different mAdMSC samples (i.e., mAdMSC pools isolated from 10 to 15 mice) were analyzed after exofucosylation by flow cytometry, showing all of them a comparable and homogeneous surface sLe^*x*^ expression (Figure 1F, left). The maximum level of sLe^*x*^ expression on mAdMSCs was achieved promptly after fucosylation, decreased to ∼65% after 24 h and reached again basal levels (similar to typical sLe^*x*^ expression on mAdMSCs) at 96 h post-treatment (Figure 1F, right).

### Comparative Analysis of the Produced Secretome by UmAdMSCs and FucmAdMSCs

To evaluate whether exofucosylation could alter mAdMSCs secretome, we performed a comparative study of the secretome released by both UmAdMSCs and FucmAdMSCs up to 72 h of serum-free cultures, analyzing a set of cytokines and chemokines involved in numerous immune pro- and anti-inflammatory processes (Turner et al., 2014). We grouped these molecules according to their recognized functional properties. Thus, we evaluated the pro-inflammatory cytokines IFNγ, IL12 (p40 and p70), IL17, IL1α, IL1β, GM-CSF, IL6, IL2, and TNFα; the anti-inflammatory cytokines IL4 and IL10; chemokines and other chemoattractant molecules such as KC (keratinocyte-derived chemokine), MIP-1α, MIP-1α, MCP-1, RANTES and eotaxin; and other cytokines or growth factors, such as G-CSF, IL3, IL5, IL9, and IL13.

**FIGURE 2:**
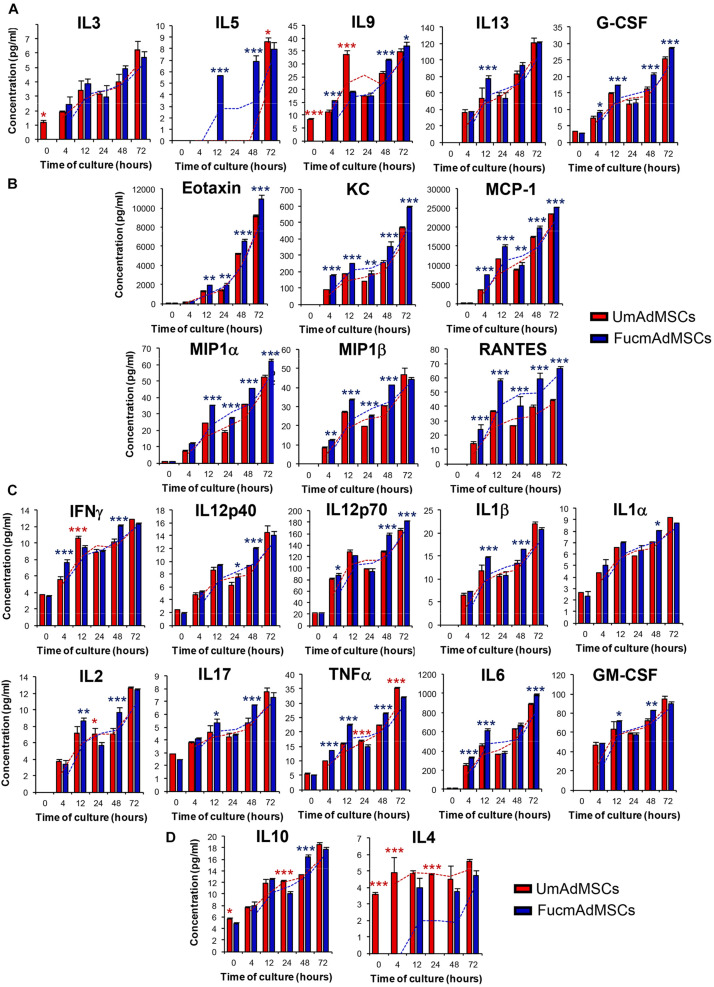
Comparison of the secretome profile of UmAdMSCs and FucmAdMSC in conditioned media obtained along the time of culture. Pooled mAdMSCs isolated from adipose tissue from several mice (*n* = 15) were treated with FTVII and the donor substrate GDP-fucose (FucmAdMSCs) or buffer alone (UmAdMSCs), allowed to adhere, re-fed with serum-free media and cultured up to 72 h. At the indicated times, conditioned media samples from UmAdMSC or FucmAdMSC cultures were collected, filtered and comparatively analyzed using the Bio-Plex Pro mouse cytokine, chemokine, and growth factor magnetic bead-based assays in a Luminex system, according to the manufacturer’s instructions. The concentration of growth factors and other regulatory cytokines **(A)**, chemokines **(B)**, pro-inflammatory cytokines **(C)**, and anti-inflammatory cytokines **(D)** secreted by UmAdMSCs (red bars) or FucmAdMSCs (blue bars) were measured. Levels of each analyte detected in the culture medium alone were used as blank. Data represent the mean ± SD from three independent experiments and were analyzed using one-way ANOVA followed by Tukey’s *post-hoc* comparison tests. Levels of each analyte was significantly up-regulated in UmAdMSCs (red asterisks) or in FucmAdMSCs (blue asterisks) at the indicated time points, *p < 0.05, **p < 0.01, or ***p < 0.001, respectively.

The production of the studied molecules followed the same pattern in the cultures of both UmAdMSCs and FucmAdMSCs, with a low production in the first hours of culture and gradually increasing onward to reach maximum values in the last studied stages (48 h and 72 h; Figures 2A–D).

For IL5 (12 h and 48 h), IL9 (4 h and 48 h), IL13 (12 h), and G-CSF (4, 12, 48, and 72 h), production was significantly higher in FucmAdMSCs compared to UmAdMSCs (Figure 2A). Notably, at all time-points, all the studied chemokines and chemoattractant molecules (eotaxin, KC, MCP-1, MIP1α, MIP1β, and RANTES) showed higher values in FucmAdMSC cultures than in UmAdMSC cultures (Figure 2B). Moreover, mostly at 48 h of culture, FucmAdMSCs also exhibited higher levels of pro-inflammatory cytokines (i.e., IFNγ, IL12p40, and p70, IL1β, IL1α, IL2, IL17, TNFα, IL6, and GM-CSF), although levels of these molecules equalized to that of UmAdMSCs at 72 h (Figure 2C); in contrast, production of anti-inflammatory cytokines, mainly IL4 (0, 4, and 24 h) but, also, IL10 (0 h and 24 h), was significantly higher in UmAdMSCs (Figure 2D).

### Comparative Evaluation of Immunomodulatory Molecules in FucmAdMSCs and UmAdMSCs With and Without Stimulation by IFNγ

**FIGURE 3:**
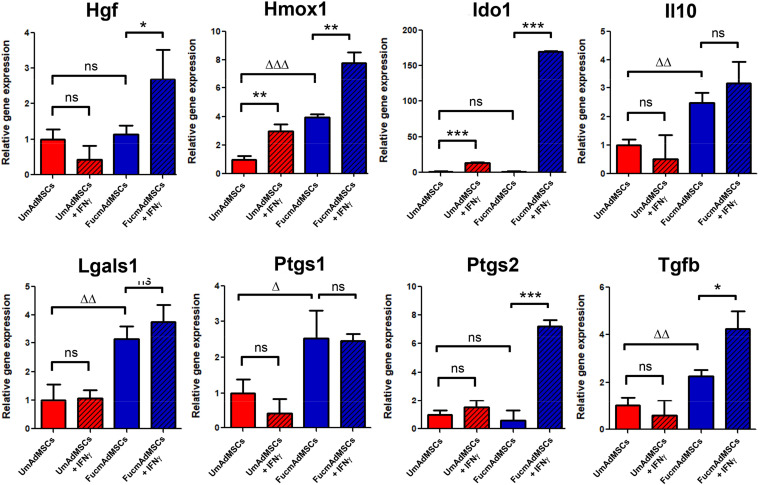
Analysis of the expression of immunomodulatory molecules in mAdMSCs by qPCR. Different pools of UmAdMSCs or FucmAdMSCs were allowed to adhere and cultured in complete medium for 24 h. Then, cells were stimulated with IFNγ or left unstimulated (negative control) for additional 24 h. Then, expression of hepatocyte growth factor (Hgf), heme-oxygenase 1 (Hmox1), indoleamine 2,3-dioxygenase (Ido1), interleukin-10 (Il10), galectin-1 (Lgals1), COX-1 (Ptgs1), COX-2 (Ptgs2), and transforming growth factor-β (Tgfb) were analyzed by qPCR. Mouse Gapdh was used as housekeeping gene to quantify and normalize the results. Fold-change gene expression was assessed by the 2^– ΔΔ*Ct*^ method. Data represent the mean ± SD from three independent experiments and were analyzed using one-way ANOVA followed by Tukey’s *post-hoc* comparison tests. Expression was significantly up-regulated in FucmAdMSCs compared to UmAdMSCs, ^ΔΔ^*p* < 0.01, ^ΔΔΔ^*p* < 0.001, or in IFNγ-stimulated mAdMSCs compared to unstimulated counterparts, *p < 0.05, **p < 0.01, and ***p < 0.001, respectively. ns: not significant.

**FIGURE 4:**
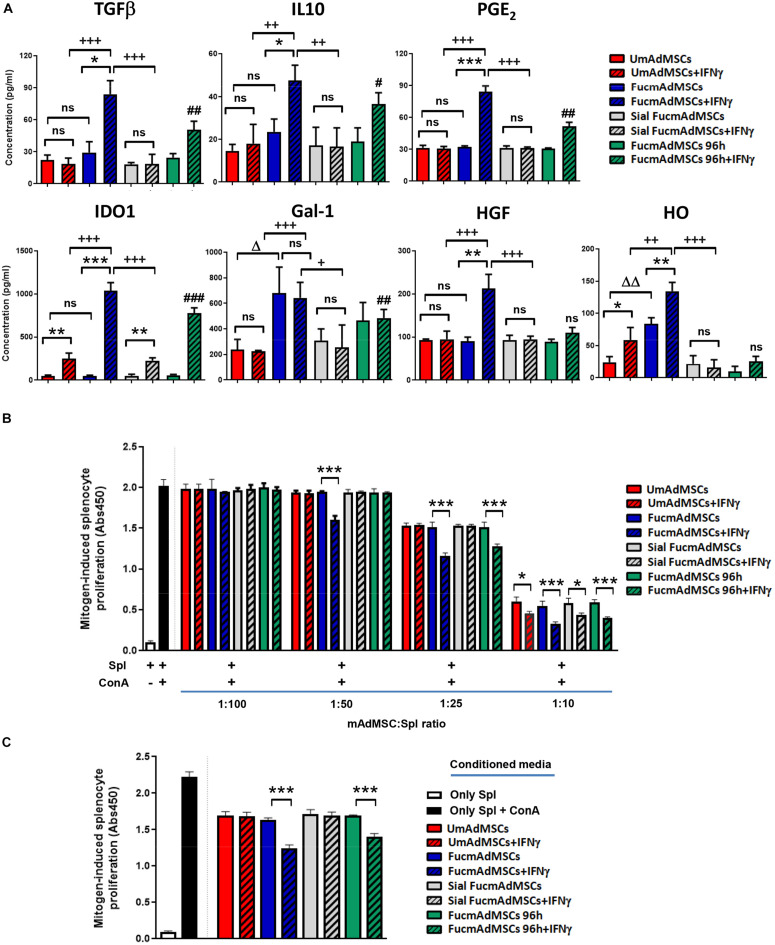
**(A)** Analysis of immunomodulatory molecule secretion in mAdMSCs by ELISA. Different pools of UmAdMSCs, FucmAdMSCs, or sialidase-treated FucmAdMSCs (Sial FucmAdMSCs) were allowed to adhere and cultured in complete medium for 24 h. After, cells were stimulated or not (negative control) with IFNγ for 24 h and cultured for additional 48 h. As control, other group of FucmAdMSCs cells were cultured for 96 h (FucmAdMSCs 96 h) before stimulation with IFNγ for 24 h. Then, levels of secreted transforming growth factor-β (TGFβ), interleukin-10 (IL10), prostaglandin E_2_ (PGE_2_), indoleamine 2,3-dioxygenase 1 (IDO1), galectin-1 (Gal-1), hepatocyte growth factor (HGF), and heme-oxygenase 1 (HO) were analyzed by ELISA. Data represent the mean ± SD from three independent experiments and were analyzed using one-way ANOVA followed by Tukey’s *post-hoc* comparison tests. Levels of each molecule was significantly up-regulated in FucmAdMSCs compared to UmAdMSCs, ^Δ^*p* < 0.05, ^ΔΔ^*p* < 0.01, or in IFNγ-stimulated mAdMSCs compared to their unstimulated counterparts, *p < 0.05, **p < 0.01, ***p < 0.001, or in IFNγ-stimulated FucmAdMSCs compared to INFγ-stimulated UmAdMSCs or compared to IFNγ-stimulated Sial FucmAdMSCs, ^+^p < 0.05, ^++^*p* < 0.01, ^+++^*p* < 0.001, or in IFNγ-stimulated FucmAdMSCs 96 h compared to IFNγ-stimulated UmAdMSCs, ^#^*p* < 0.05, ^##^*p* < 0.01, and ^###^*p* < 0.001, respectively. ns: not significant. **(B)** Concanavalin A-stimulated splenocytes were co-cultured with different ratios of UmAdMSCs, FucmAdMSCs, Sial FucmAdMSCs, and FucmAdMSCs 96 h, previously treated or not with IFNγ for 24 h. After 3 days of co-cultures, splenocyte proliferation was determined by BrdU incorporation. Data represent the mean ± SD from three independent experiments and were analyzed using one-way ANOVA followed by Tukey’s *post-hoc* comparison tests. Splenocyte proliferation in presence of IFNγ-stimulated mAdMSCs was significantly inhibited compared to their unstimulated counterparts at each specific MSC:splenocyte ratio, *p < 0.05, ***p < 0,001, respectively. Spl, splenocyte, ConA, concanavalin A. **(C)** Additional mitogenic experiments were performed with ConA-stimulated splenocytes in presence of conditioned media obtained from MSC:splenocyte co-cultures at 1:25 ratio. After 3 days of culture, splenocyte proliferation was measured by BrdU incorporation. Data represent the mean ± SD from three independent experiments and were analyzed using one-way ANOVA followed by Tukey’s *post-hoc* comparison tests. Splenocyte proliferation in presence of IFNγ-stimulated mAdMSC:splenocyte conditioned media was significantly inhibited compared to proliferation levels obtained with conditioned media from unstimulated MSC:splenocyte co-cultures, ***p < 0,001.

Apart from measurements of protein levels, we also performed qPCR and ELISAs to evaluate the impact of exofucosylation on gene expression of key immunomodulatory molecules in AdMSCs cultured in complete medium. We evaluated comparatively the produced transcripts in UmAdMSCs and FucmAdMSCs under steady-state culture and following treatment with IFNγ for 24 h to mimic conditions found in a pro-inflammatory milieu. We observed that, except for Hgf, Ido1 and Ptgs2 (COX2), which showed no transcript level differences between unstimulated UmAdMSCs and FucmAdMSCs, the other studied molecules, including Hmox1 (heme-oxygenase), Il10, Lgals1 (galectin-1), Ptgs1 (COX1), and Tgfb, showed significantly higher RNA levels in unstimulated FucmAdMSCs than in unstimulated UmAdMSCs or control mAdMSCs (i.e., GDP-fuc-mAdMSCs and FTVII-mAdMSCs; Figure 3 and Supplementary Figure 2A). With IFNγ-stimulation of UmAdMSCs or control mAdMSCs, Hmox1 and Ido1 levels increased, but there was no increase in the other transcripts. In FucmAdMSCs treated with IFNγ, there were increases in Hgf, Hmox1, and Tgfb transcripts, with markedly increased levels of Ptgs2 and Ido1 (12-fold and 154-fold, respectively; Figure 3). In addition, although Ido1 production increased in IFNγ-stimulated UmAdMSCs, control mAdMSCs and FucmAdMSCs, the increase in the latter was much higher. However, Il10, Lgals1 and Ptgs1 transcripts did not exhibit a significant increase in IFNγ-treated FucmAdMSCs compared to unstimulated counterparts, suggesting that, following exofucosylation, expression of these genes are not further affected by IFNγ exposure. Nevertheless, when the effects of IFNγ-stimulation were evaluated regarding protein expression, TGFβ, IL10, PGE_2_, IDO1, Gal-1, HGF, and heme-oxygenase (HO) values were significantly increased after 72 h of culture in FucmAdMSCs compared to UmAdMSCs and control mAdMSCs (Figure 4A and Supplementary Figure 2B). Importantly, this increased secretion of soluble anti-inflammatory molecules was significantly abrogated by digestion of sLe^*X*^-decorated HCELL molecules following sialidase treatment of FucmAdMSCs (i.e., Sial FucmAdMSCs, Figure 4A).

As the above described results indicated that the process of fucosylation induces changes in properties of mAdMSCs, we comparatively evaluated various other characteristics and capabilities of UmAdMSCs and FucmAdMSCs in an attempt to elucidate other phenotypic differences.

### UmAdMSC and FucmAdMSC-Induced Inhibition of Mitogen-Stimulated Splenocyte Proliferation

To assess whether HCELL expression functionally affects the immunosuppressive properties of mAdMSCs, we analyzed the capacity of UmAdMSCs and FucmAdMSCs, previously treated or not with IFNγ for 24 h, to inhibit mitogen-stimulated splenocyte proliferation. We observed that concanavalin A (ConA)-stimulated splenocyte proliferation was significantly decreased when increasing ratios of mAdMSCs (from 1:100 to 1:10 MSC:splenocyte) were added to the cultures (Figure 4B). However, IFNγ-treated FucmAdMSCs displayed a significant suppression of splenocyte proliferation from 1:50 MSC:splenocyte ratio compared to their untreated counterparts, whereas IFNγ-treated UmAdMSCs or IFNγ-treated Sial FucmAdMSCs required a higher ratio MSC:splenocyte (i.e., 1:10) to show a significant immunosuppressive effect on ConA-splenocyte proliferation (Figure 4B).

To further investigate the relevance of mAdMSC secretome on their observed immunosuppressive effect, we performed additional mitogenic assays using conditioned media from MSC:splenocyte co-cultures (1:25 ratio). Supernatants obtained from co-cultures containing IFNγ-stimulated FucmAdMSCs showed a significant inhibition of mitogen-induced splenocyte proliferation compared to that observed with UmAdMSCs or Sial FucmAdMSC conditioned media, and to an extent comparable to that found in the continuous presence of mAdMSCs (Figure 4C).

### Morphology and Cell Proliferation of UmAdMSCs and FucmAdMSCs

**FIGURE 5:**
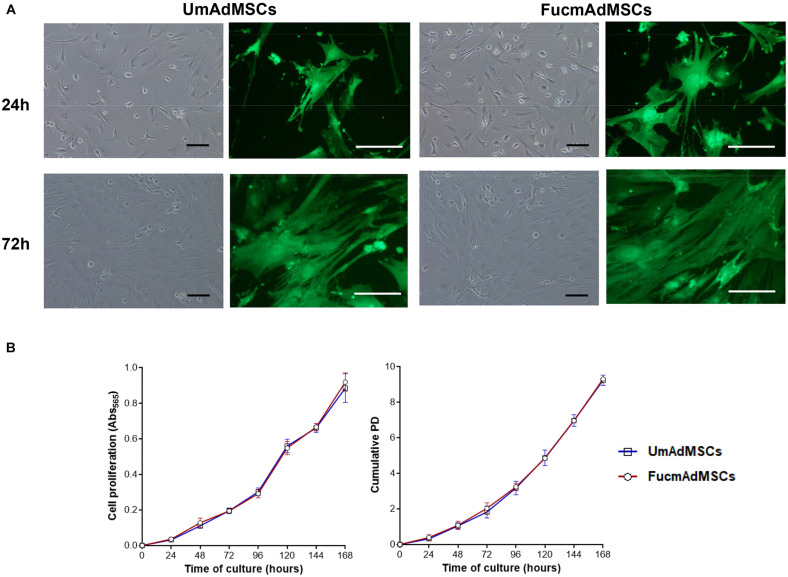
Analysis of the cell morphology and proliferation of UmAdMSCs and FucmAdMSCs. **(A)** UmAdMSCs and FucmAdMSCs were cultured up to 72 h to analyze their typical MSC morphology. Phase-contrast and fluorescence microscopy images at higher magnification of GFP-expressing mAdMSCs are shown. Scale bar: 100 μm. **(B)** UmAdMSCs and FucmAdMSCs were cultured up to 168 h to analyze their cell proliferation rates using the standard colorimetric MTT assay (**B**, left) or calculating their cumulative population doubling (PD; **B**, right) using the formula specified in the Materials and Methods section.

In order to analyze whether fucosylation could affect MSC morphology, UmAdMSCs and FucmAdMSCs were cultured up to 72 h. Both cell types displayed similar plastic adherence, a spindle-shape and a fibroblast-like morphology, typical of MSCs (Figure 5A). In addition, proliferation assays were carried out with the two cell types using the standard colorimetric MTT assay and cumulative PD calculation up to 7 days of culture. Both UmAdMSCs and FucmAdMSC exhibited significantly similar exponential cell growth curves and cumulative PD (*p* > 0.05; Figure 5B).

### FTVII-Treatment of mAdMSCs Does Not Alter Cell Immunophenotype or Multilineage Differentiation Efficiency

**FIGURE 6:**
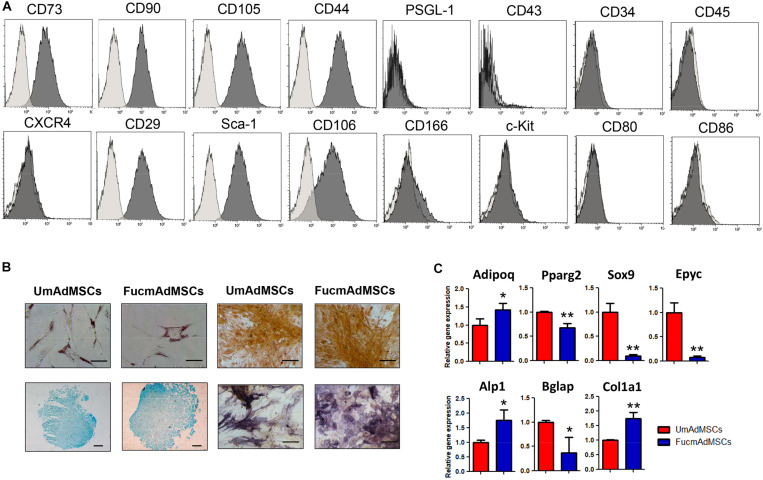
Fucosylation of mAdMSCs does not affect MSC immunophenotype or multilineage differentiation properties. **(A)** mAdMSCs expressed typical MSC surface markers such as CD73, CD90, CD105, CD44, CD29, Sca-1, CD106, and CD166, and displayed a negligible or negative expression of CD34, CD45, CXCR4, c-Kit, CD80 and CD86, or other E-selectin ligands such as PSGL-1 or CD43. Control isotype antibodies staining are represented as light gray histograms. Representative results from *n* = 3 independent experiments are shown. **(B)** UmAdMSCs or FucmAdMSCs were cultured in specific media to evaluate their *in vitro* differentiation capacity into the adipogenic, osteogenic and chondrogenic lineages. Adipogenic differentiation was evaluated by staining of cytoplasmic lipid-laden vacuoles with Oil Red O (adipocytes; **B**, top left), whereas osteogenic commitment was analyzed by staining of calcium deposits with Alizarin Red (**B**, top right) or alkaline phosphatase activity with BCIP-NBT (**B**, bottom right). Chondrogenic differentiation was evaluated by staining of acidic mucopolysaccharides with Alcian blue (**B**, bottom left). Images shown are representative of three independent differentiation assays. Scale bar: 200 μm. **(C)** Expression of adiponectin (Adipoq), PPARγ2 (Pparg2), SOX9 (Sox9), epiphycan (Epyc), alkaline phosphatase (Alp1), osteocalcin (Bglap), and collagen type I (α1 chain; Col1a1) were analyzed by qPCR. Mouse Gapdh was used as housekeeping gene to quantify and normalize the results. Fold-change gene expression was assessed by the 2^– ΔΔ*Ct*^ method. Data represent the mean ± SD from three independent experiments and were analyzed using one-way ANOVA followed by Tukey’s *post-hoc* comparison tests. Expression was significantly up-regulated or down-regulated in FucmAdMSCs compared to UmAdMSCs, *p < 0.05, **p < 0.01, respectively.

As determined by flow cytometry, mAdMSCs expressed typical MSC surface markers such as CD73, CD90, CD105, CD44, CD29 (β1 integrin), Sca-1, CD106 (VCAM-1), and CD166 (ALCAM-1), together with low or no expression of the hematopoietic markers CD34, CD45, CXCR4, c-Kit, CD80 and CD86, or other E-selectin ligands such as PSGL-1 or CD43 (Figure 6A). To further evaluate whether exofucosylation could modify the immunophenotypic profile of mAdMSCs, we analyzed the expression of the positive MSC markers up to 72 h post-treatment and in different mAdMSC culture passages (from passage 2 up to passage 5). Despite a slight up- or down-regulation of some markers, presumably due to differences in cell confluence along the time of culture, FucmAdMSCs displayed no significant different percentage of positive expression and mean fluorescence intensity values (MFI) for these markers compared to UmAdMSCs (*p* > 0.05; Supplementary Table 3).

Moreover, we evaluated whether FTVII-treatment could affect the multilineage differentiation potential of mAdMSCs. For this purpose, UmAdMSCs, and FucmAdMSCs were cultured in either adipogenic, osteogenic, or chondrogenic differentiation media. After specific qualitative staining, FucmAdMSCs showed no apparent visual differences in the formation of cytoplasmic lipid-laden vacuoles (adipocytes; Figure 6B, top left), in the levels of matrix calcium deposits or alkaline phosphatase activity (osteoblasts; Figure 6B, top right and bottom right, respectively), and neither in expression of acidic mucopolysaccharides (chondroblasts; Figure 6B, bottom left). However, qPCR analyses revealed a significantly different expression of several adipocyte –, i.e., adiponectin (Adipoq) and PPARγ2 (Pparg2)-, chondroblast –, i.e., SOX9 (Sox9) and epiphycan (Epyc)- and osteoblast –, i.e., alkaline phosphatase (Alp1), osteocalcin (Bglap) and collagen type I (Col1a1)-lineage-specific markers between UmAdMSCs and FucmAdMSCs (Figure 6C). These results suggest that although FucmAdMSCs are able to differentiate efficiently to adipocyte, osteoblast and chondroblast cell lineages, exofucosylation could affect the transition or length of the different stages during MSC multipotent differentiation.

### Comparative Evaluation of the Migratory Capacities of UmAdMSCs and FucmAdMSCs

**FIGURE 7:**
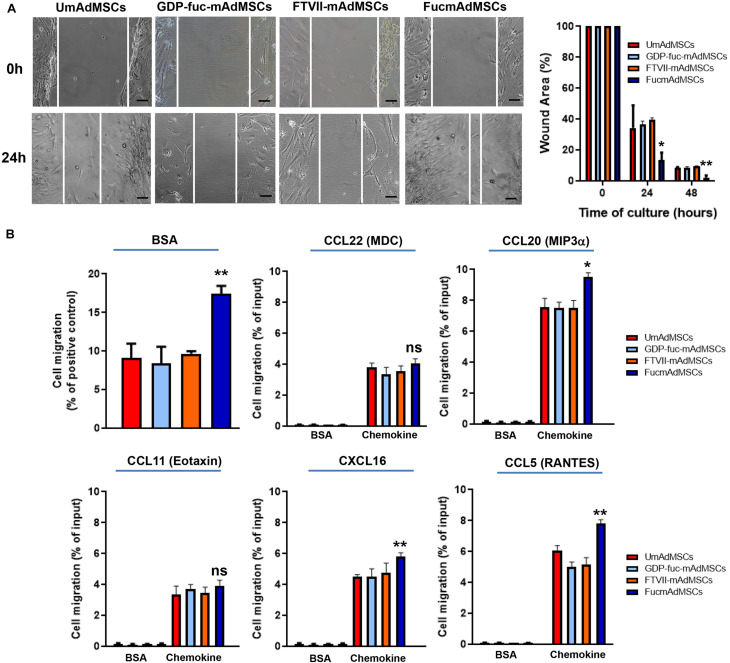
Exofucosylation increases the migratory capacity of mAdMSCs. **(A)** Migration of UmAdMSCs, GDP-fuc-mAdMSCs, FTVII-mAdMSCs, and FucmAdMSCs were analyzed by wound-healing assays. After obtaining confluent monolayers of both types of cells, a scratch was performed. Subsequently, images of the scratched area were captured after 24 h or 48 h of wounding. Then, the wound areas at the indicated times were calculated in relative to the total wound area at 0 h of the same wound spot. Representative images of initial scratch (0 h) or after 24 h are shown. Each condition was analyzed in triplicate. Wound area in FucmAdMSC cultures was significantly lower, *p < 0.05, **p < 0.01, respectively. Scale bar: 100 μm. **(B)** Migration assays were carried out using a transwell system. UmAdMSCs, GDP-fuc-mAdMSCs, FTVII-mAdMSCs, and FucmAdMSCs were allowed to migrate toward MesenCult medium containing 30% FBS (positive control of cell migration) or 0.3% BSA (spontaneous migration) for 24 h at 37°C. Also, mAdMSC-induced migration in response to the indicated pro-inflammatory chemokines was analyzed after incubation for 6 h at 37°C. Extent of cell migration to the lower chamber was quantified. FucmAdMSC migration was significantly increased, *p < 0.01, **p < 0.01, respectively, according to one-way ANOVA followed by Tukey’s *post-hoc* comparison tests. Each condition was analyzed in triplicate. Data were expressed as mean percentages ± SD of migrated cells related to the positive control (FBS) or input (100% of migration).

To evaluate migratory capacity, scratch was performed in confluent cultures of UmAdMSCs, GDP-fuc-mAdMSCs, FTVII-mAdMSCs, or FucmAdMSCs, and the scratched area was measured after 24 h and 48 h of culture (Figure 7A). At both times, the scratched area was significantly smaller in FucmAdMSCs than in UmAdMSCs or control mAdMSCs (Figure 7A). FucmAdMSCs also exhibited significantly higher spontaneous migratory capabilities than UmAdMSCs or control mAdMSCs in transwell assays (BSA, Figure 7B, top left), and in response to several pro-inflammatory chemokines [i.e., CCL20 (MIP3α), CXCL16 and CCL5 (RANTES)] (Figure 3B), confirming what has been reported using other approaches (Kocher et al., 2001; Sackstein et al., 2008; Abdi et al., 2015; Dykstra et al., 2016).

## Discussion

Mesenchymal stromal cells currently represent the most promising cell type for cell therapy largely due to their anti-inflammatory and immunomodulatory properties (Caplan, 2007; Chamberlain et al., 2007). However, there are many unresolved questions regarding the therapeutic applications of these cells.

To date, four E-selectin ligands have been identified: cutaneous lymphocyte antigen (CLA, sialofucosylated PSGL-1 glycoform), E-selectin ligand-1 (ESL-1), CD43E (sialofucosylated CD43 glycoform), and hematopoietic cell E-selectin/L-selectin ligand (HCELL, sialofucosylated CD44 glycoform), whose expression is restricted to leukocytes and hematopoietic stem cells (Hidalgo et al., 2007; Merzaban et al., 2011; Silva et al., 2017). However, MSCs do not express any sLe^*x*^-decorated E-selectin ligand, which results in the lack of specific migration of these cells to inflamed tissues, since E-selectin, heavily expressed in the endothelial cells on the sites of inflammation after stimulation by pro-inflammatory cytokines (Schweitzer et al., 1996; Jung et al., 1997; Yao et al., 1999; Chong et al., 2004), is necessary for cell transmigration. In this respect, Sackstein and colleagues demonstrated that both extracellular (Sackstein et al., 2008; Abdi et al., 2015; Pachón-Peña et al., 2016), and intracellular fucosylation of CD44, resulted in increased MSC migration to bone or to damaged tissues (Abdi et al., 2015; Dykstra et al., 2016).

Apart from differences in E-selectin binding, no other differences have been reported to date between FTVII-modified and unmodified MSCs. In agreement with our current results, Chou et al. found no changes in differentiation between exofucosylated and native bone marrow-derived MSCs, nor differences in cell proliferative rate after ten passages of cultures (Chou et al., 2017). However, our present results indicate that there are important differences between the pattern of soluble molecules secretion of UmAdMSCs and FucmAdMSCs, a feature not described previously. Other studies reported differences between MSC secretome largely related with the source of cells (Hsiao et al., 2012; Hsieh et al., 2013; Amable et al., 2014; Du et al., 2016), isolation methods, culture conditions and detection assays as well as due to species-specific variations (Chen et al., 2008; Park et al., 2009; Rodriguez et al., 2015; Hirose et al., 2018). Recently, it has been described that other type of cells such as memory T regulatory cells (Tregs), the vast majority of which heavily express sLe^*x*^ and exhibit strong transendothelial migration ability, but not naïve Tregs which show lower levels of sialofucosylated E-selectin ligands (Donnelly et al., 2018), produce differentially IL17 and IFNγ (Koenen et al., 2008; Booth et al., 2010).

After serum withdrawal (experimental conditions shown in Figure 2), FucmAdMSCs produce significantly more pro-inflammatory cytokines (i.e., IFNγ, IL12p40/p70, IL1β, IL2, IL6, GM-CSF, IL17, and TNFα) and other regulatory cytokines and growth factors (i.e., G-CSF, IL5, IL9, and IL13) than UmAdMSC, a fact that has been related with activated MSCs (Rodriguez et al., 2015). It is important to remark that it has been previously addressed that MSCs are sensitive to serum or nutrient deprivation, a situation they may encounter in an ischemic microenvironment (Nuschke et al., 2016). Survival mechanisms up-regulated in MSCs in response to starvation include, among others, activation of autophagy pathways, and some phenotypic changes such as an increased differentiation capacity (Pantovic et al., 2013; Nuschke et al., 2014). However, to date it has not been described in the literature how lack of nutrients may affect the secretory profile of MSCs. Other authors have described a similar activation pattern after exposing MSCs to different pro-inflammatory stimuli such as IFNγ, TNFα, IL-1β, lipopolysaccharide or hypoxia, a process which depends on the nuclear factor-kappa B (NF-κB) signaling pathway (Crisostomo et al., 2008). On the other hand, increased production of some of these pro-inflammatory cytokines by extravasated FucmAdMSCs could favor, in a paracrine fashion, the migratory capacity of other circulating FucmAdMSCs through its local effect on endothelium. It is well known that TNFα and IL1, IL12, IL-6, IL-13, IFNγ, or IL17A, alone or in synergy with TNFα, differentially up-regulate the expression of several adhesion molecules on microvascular endothelial cells at sites of tissue injury or inflammation, in particular, E-selectin, VCAM-1, and ICAM-1 (Pober et al., 1986a, b; Bevilacqua et al., 1987; Dustin and Springer, 1988; Bochner et al., 1995; Vestweber and Blanks, 1999; Stocker et al., 2000; Strasly et al., 2001; Chiu et al., 2007; Sackstein, 2009; Griffin et al., 2012). Also, G-CSF induces endothelial expression of E-selectin and ICAM-1 through activation of p38MAPK (Fuste et al., 2004). On the other hand, some pro-inflammatory cytokines produced by MSCs have indirectly immunosuppressive properties. For instance, IL2 promotes the development of CD4^+^CD25^+^FoxP3^+^ Tregs (Malek et al., 2000; Malek, 2003; Burchill et al., 2007), that represent an immunomodulatory T cell specialized subset able to neutralize the development of immune-mediated damage and to promote repair and regeneration of affected target-organs in systemic immune diseases (Sakaguchi et al., 1995; Gatza et al., 2008). Also, IL9 has been described to enhance the immunosuppressive function of Tregs (Elyaman et al., 2009; Eller et al., 2011), and IL13 inhibits pro-inflammatory cytokine production and shares anti-inflammatory properties with IL4 (de Waal Malefyt et al., 1993; Minty et al., 1993).

Previous studies reported that MSCs isolated from different sources secrete numerous chemokines, i.e., SDF-1/CXCL12, MCP-1/CCL2, MCP-2/CCL8, MCP-3/CCL7, eotaxin/CCL11, GCP-2/CXCL6, KC/CXCL1, IP10/CXCL10, and others (Ma et al., 2014; Rodriguez et al., 2015). In the present work, we show that FucmAdMSCs secrete increased levels of several chemokines (i.e., eotaxin, KC, MCP-1, MIP1α, MIP1β, and RANTES) compared to their unmodified counterparts. Ren et al. (2008) reported that after stimulation with various pro-inflammatory cytokines, MSCs increase their ability to secrete high levels of chemokines that attract neighboring inflammatory immune cells (i.e., T cells) into proximity with MSCs, suppressing T cell responses by production of nitric oxide. On the other hand, we have found in standard conditions of culture (i.e., in presence of nutrients and growth factors) that expression of the anti-inflammatory cytokine IL10 and other immunosuppressive factors, such as PTGS1 (COX1), HO, and TGFβ were higher in FucmAdMSCs than in UmAdMSCs. Remarkably, after IFNγ-stimulation, the expression of these molecules at protein level, particularly that of IDO1, increased enormously in FucmAdMSCs, as previously reported (Chon et al., 1996; Ren et al., 2009), and retained the ability to secrete significant higher levels of other anti-inflammatory molecules (i.e., TGFβ, IL10, PGE_2_, Gal-1, HGF, and HO) that mediate their observed higher anti-proliferative effects in a pro-inflammatory milieu even after they have already lost HCELL expression (i.e., FucmAdMSCs 96 h; Figures 4A–C). Thus, and in agreement with results of Ren et al. (2008), our findings also suggest that a higher production of chemokines and anti-inflammatory molecules acquired by mAdMSCs after their FTVII-treatment could favor a local negative feedback loop, suppressing the inflammatory immune response in the vicinity of MSCs.

Our results also indicate that exofucosylated mAdMSCs show a higher spontaneous migration capability and in response to different inflammatory chemokines than their unmodified counterparts. Given the well-known role of chemokines in the recruitment and homing of immune cells to tissues (Moser and Loetscher, 2001), our findings suggests that FucmAdMSCs, apart from their enhanced tethering and rolling contacts on E-selectin-bearing endothelial cells, could possess some additional advantages in their chemokine-mediated homing to inflamed or injured tissues after systemic administration.

First question emerging from our study is how the absence or presence of nutrients in a concrete microenvironment could affect tissue-resident mAdMSC phenotype and function regarding secretion of different soluble mediators. In presence of nutrients and growth factors, which are *in vitro* provided by the serum of the culture medium, mAdMSCs exhibited an anti-inflammatory phenotype, mainly in FucmAdMSCs stimulated with IFNγ, whilst starvation induced a switch from an anti-inflammatory FucmAdMSC phenotype to another one that mainly secreted pro-inflammatory cytokines, growth factors and chemokines. Accordingly, the nutritional deficiency or abundance that a certain tissue presents depending on factors such as anatomical location or blood supply, can greatly affect the mAdMSCs functional properties and therefore their subsequent immunomodulatory response.

Secondly, it has not been previously addressed how enzymatic exofucosylation could result in this distinct, specific behavior and how it can also affect the immunomodulatory properties of FucmAdMSCs. In this respect, it is possible that CD44 and its sialofucosylated form HCELL could establish *cis*-interactions resulting in different cellular behavior of FucmAdMSCs compared to UmAdMSCs, interacting with different ligands or establish interactions of different intensity and/or time length. Although we have not studied these mechanisms in detail in this work, we believe that this difference [i.e., an additional fucose residue added after exofucosylation in α(1,3) of N-acetylglucosamine of terminal tetrasaccharyde sLe^*x*^] (Sackstein, 2009) could be sufficient to alter the molecule affinity resulting in different signaling and activation. In this respect, CD44v expressed on tumor cells but not CD44s expressed on MSCs can form molecular clusters (Sleeman et al., 1996). Recently, we demonstrated that an altered adhesion affected importantly the behavior of bone marrow human MSCs (Alfaro and Zapata, 2018). Taking all our results together, the changes in the cellular behavior induced by exofucosylation, ultimately results in a functional polarization of the MSCs toward an inhibitory, scarce pro-inflammatory phenotype.

In summary, these results provide new perspectives on the functional features and mechanisms of action of FucmAdMSCs that could have important therapeutic consequences, highlighting the specific secretome observed in FucmAdMSCs and its possible *in vivo* relevance in a variety of preclinical studies.

## Conclusion

This study represents an in-depth phenotypical and functional direct comparison between native mAdMSCs and mAdMSCs modified by FTVII-exofucosylation, an enzymatic method to specifically induce the transient surface expression of the E-selectin ligand HCELL. Compared to their unmodified counterparts, fucosylated mAdMSCs display a consistent increased, although transient, expression of HCELL, an augmented production of growth factors, chemokines and other chemoattractants and regulatory molecules, a higher expression/secretion of several anti-inflammatory molecules and remarkably an increased migratory and immunosuppressive properties, even after they have lost HCELL expression. Collectively, our findings suggest that fucosylation of MSCs is not only a suitable strategy to favor the recruitment of systemically infused-MSCs to the damaged tissues but also this surface glycan engineering converts MSCs into a more potent immunomodulatory/anti-inflammatory cell-based product for the treatment of a variety of autoimmune, inflammatory and degenerative diseases.

## Data Availability Statement

The original contributions presented in the study are included in the article/Supplementary Material, further inquiries can be directed to the corresponding author/s.

## Ethics Statement

All experimental animal procedures were approved by the Institutional Animal Care and Use Committee of University of Murcia and Fundación Instituto de Investigacion Sanitaria Fundación Jiménez Diaz and performed according to the established guidelines (protocols numbers A13150201 and PROEX084/16).

## Author Contributions

DG-B, MG-A, AZ, DG-O, RS, and JM designed the study. DG-B, AG-G, and MG-A performed all the experiments. DG-B, AG-G, AG-H, MB, and MG-A analyzed data. RS developed the conditions for surface fucosylation of live cells and provided essential protocols. All authors contributed to manuscript revision, read, and approved the submitted version.

## Conflict of Interest

According to National Institutes of Health policies and procedures, the Brigham and Women’s Hospital has assigned intellectual property rights regarding cell surface glycan engineering to RS, and RS has licensed portions of this technology to an entity he has founded (Warrior Therapeutics, LLC), to BioTechne, Inc., and to Mesoblast LTD. RS’s ownership interests were reviewed and are managed by the Brigham and Women’s Hospital and Partners HealthCare in accordance with their conflict of interest policy. DG-O is member of the Advisory Board of TiGenix S.A.U. MG-A and DG-O have applied for a patent related to Cx401 and Cx601 titled “Identification and isolation of multipotent cells from non-osteochondral mesenchymal tissue” (WO 2006/057649). DG-O and MG-A are shareholders of Biosurgery, an educational company providing services to Takeda. The remaining authors declare that the research was conducted in the absence of any commercial or financial relationships that could be construed as a potential conflict of interest.
