# Supplementary material for: Exofucosylation of Adipose Mesenchymal Stromal Cells Alters Their Secretome Profile
Source: Front Cell Dev Biol. 2020 Nov 26;8:584074. doi: 10.3389/fcell.2020.584074 (PMC7726227; doi:10.3389/fcell.2020.584074)
Supplement: Supplementary file 1 [file Data_Sheet_1.PDF]

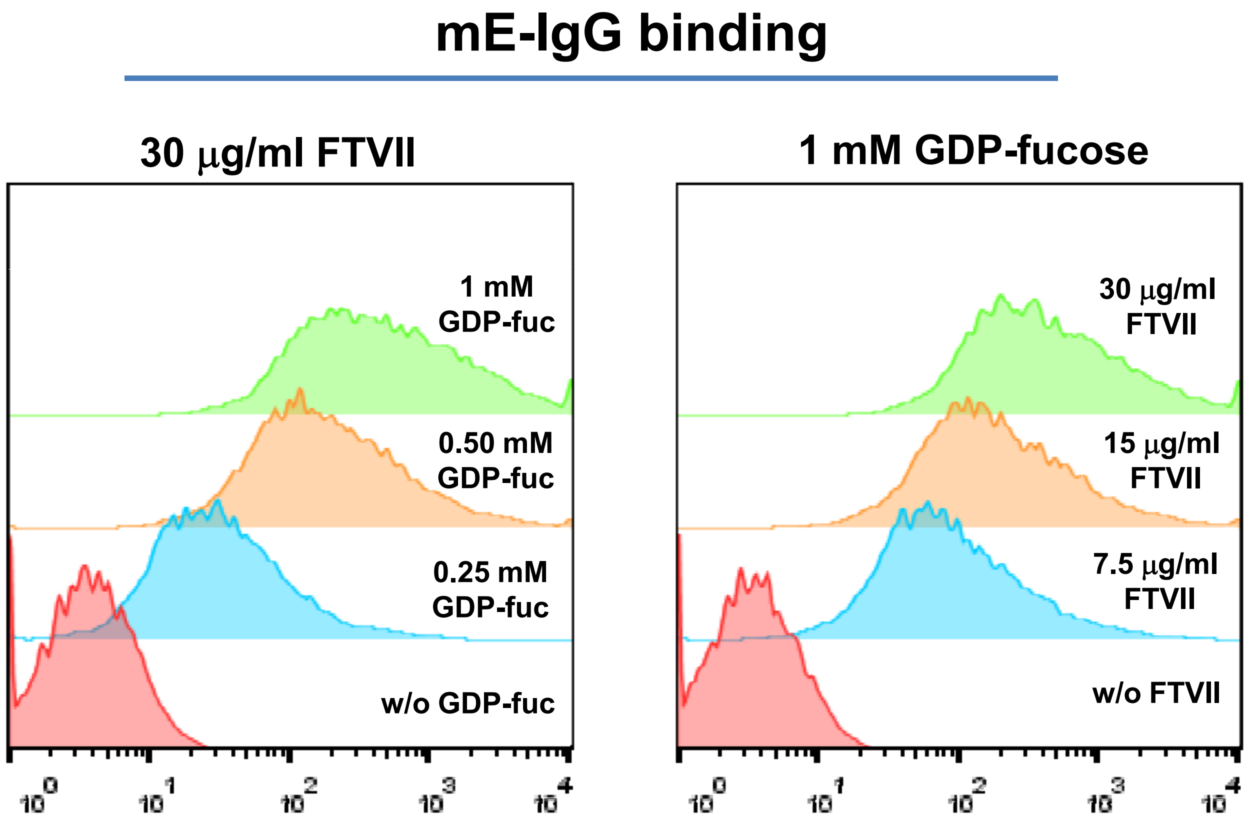

Supplementary Figure 1. Optimization of exofucosylation protocol on mAdMSCs. mAdMSCs were exofucosylated using a constant concentration of FTVII (30 µl/ml) and decreasing concentrations of GDP-fucose (left) or a constant concentration of GDP-fucose (1 mM) and decreasing concentrations of FTVII (right), and analyzed for Ca^{2+}-dependent binding of mE-IgG by flow cytometry. Representative histograms of three independent experiments are shown.
