# Supplementary material for: Exofucosylation of Adipose Mesenchymal Stromal Cells Alters Their Secretome Profile
Source: Front Cell Dev Biol. 2020 Nov 26;8:584074. doi: 10.3389/fcell.2020.584074 (PMC7726227; doi:10.3389/fcell.2020.584074)
Supplement: Supplementary file 2 [file Data_Sheet_2.PDF]

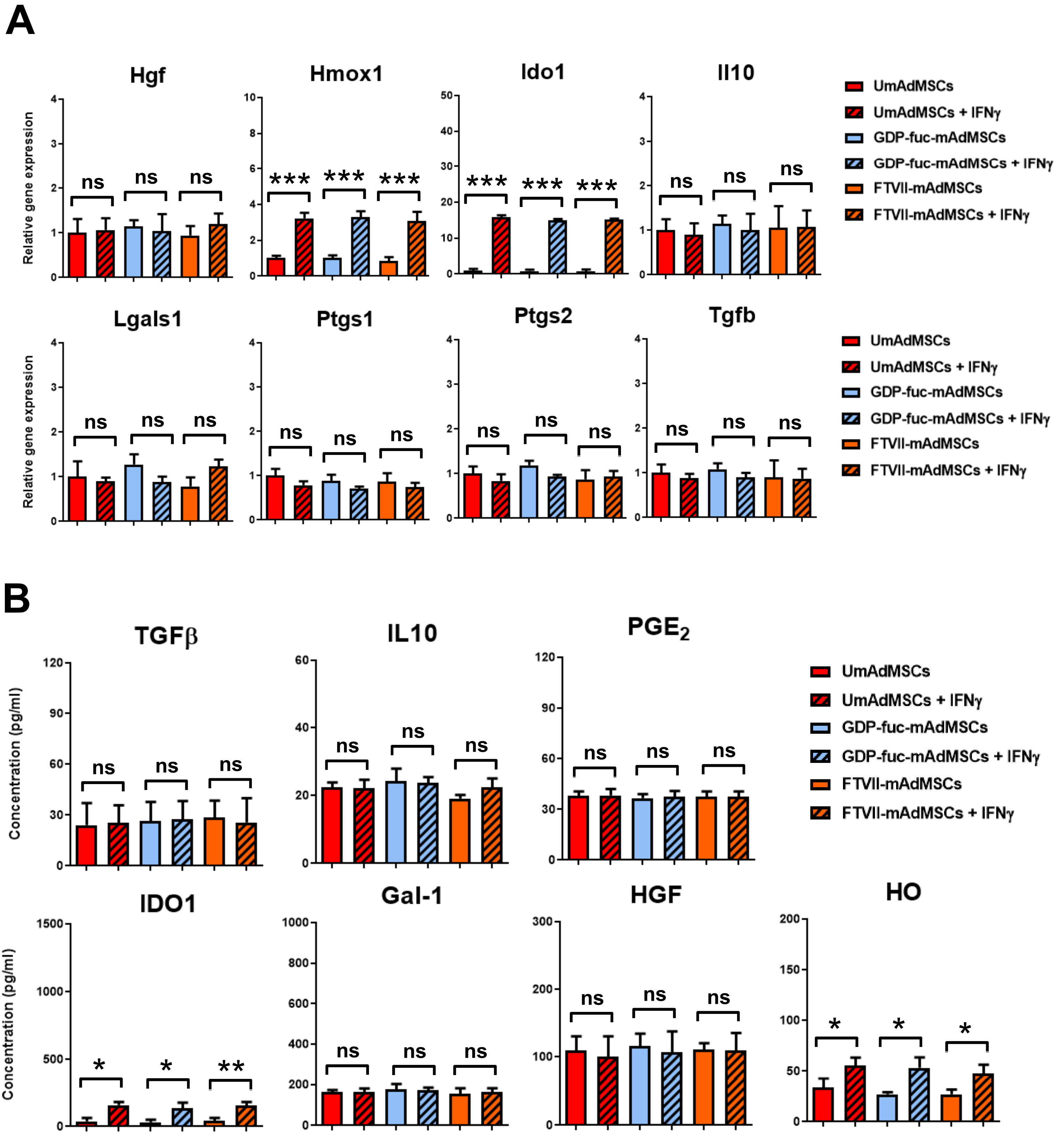

Supplementary Figure 2. Analysis of the expression of immunomodulatory molecules in mAdMSCs in presence or absence of IFNγ stimulation. **(A)** Expression of hepatocyte growth factor (Hgf), heme-oxygenase 1 (Hmox1), indoleamine 2,3-dioxygenase 1 (Ido1), interleukin-10 (Il10), galectin-1 (Lgals1), COX-1 (Ptgs1), COX-2 (Ptgs2) and transforming growth factor-β (Tgfb) at transcript level were analyzed by qPCR. Mouse Gapdh was used as housekeeping gene to quantify and normalize the results. Fold-change gene expression was assessed by the $2^{-\Delta\Delta Ct}$ method. **(B)** Expression of TGFβ, IL10, PGE_2, IDO, Gal-1, HGF and HO at protein level were analyzed by ELISA. All data represent the mean ± SD from three independent experiments and were analyzed using one-way ANOVA followed by Tukey's post-hoc comparison tests. Expression at transcript or protein levels was significantly upregulated in IFNγ-stimulated mAdMSCs compared to unstimulated counterparts, *p<0.05, **p<0.01, ***p<0.001, respectively. ns: not significant.
