# Supplementary material for: Exofucosylation of Adipose Mesenchymal Stromal Cells Alters Their Secretome Profile
Source: Front Cell Dev Biol. 2020 Nov 26;8:584074. doi: 10.3389/fcell.2020.584074 (PMC7726227; doi:10.3389/fcell.2020.584074)
Supplement: Supplementary file 3 [file Data_Sheet_3.PDF]

Analyte	Description	FI	FI - Bkgd	Std Dev	%CV	Conc in Range	Obs Conc
Mo IL-1a (53)	Reagent Name: Background0	22,50	22,50	0,71	3,14		
Mo IL-1a (53)	UmAdMSCs 0h replicate 1	27,50	5,00	0,71	2,57	2,63	2,63
Mo IL-1a (53)	UmAdMSCs 0h replicate 2	27,50	5,00	2,12	7,71	2,63	2,63
Mo IL-1a (53)	UmAdMSCs 0h replicate 3	27,50	5,00	0,71	2,57	2,63	2,63
Mo IL-1a (53)	UmAdMSCs 4h replicate 1	30,50	8,00	2,12	6,96	3,57	3,57
Mo IL-1a (53)	UmAdMSCs 4h replicate 2	33,30	10,80	1,77	5,32	4,36	4,36
Mo IL-1a (53)	UmAdMSCs 4h replicate 3	33,50	11,00	0,71	2,11	4,43	4,43
Mo IL-1a (53)	UmAdMSCs 12h replicate 1	33,50	11,00	0,71	2,11	4,43	4,43
Mo IL-1a (53)	UmAdMSCs 12h replicate 2	74,00	51,50	43,84	59,24	13,60	13,60
Mo IL-1a (53)	UmAdMSCs 12h replicate 3	42,50	20,00	0,71	1,66	6,77	6,77
Mo IL-1a (53)	UmAdMSCs 24h replicate 1	37,50	15,00	2,12	5,66	5,51	5,51
Mo IL-1a (53)	UmAdMSCs 24h replicate 2	38,00	15,50	0,00	0,00	5,64	5,64
Mo IL-1a (53)	UmAdMSCs 24h replicate 3	41,00	18,50	1,41	3,45	6,40	6,40
Mo IL-1a (53)	UmAdMSCs 48h replicate 1	47,50	25,00	3,54	7,44	7,97	7,97
Mo IL-1a (53)	UmAdMSCs 48h replicate 2	43,50	21,00	2,12	4,88	7,02	7,02
Mo IL-1a (53)	UmAdMSCs 48h replicate 3	41,50	19,00	0,71	1,70	6,53	6,53
Mo IL-1a (53)	UmAdMSCs 72h replicate 1	51,00	28,50	4,24	8,32	8,77	8,77
Mo IL-1a (53)	UmAdMSCs 72h replicate 2	55,50	33,00	4,95	8,92	9,77	9,77
Mo IL-1a (53)	UmAdMSCs 72h replicate 3	48,00	25,50	4,24	8,84	8,08	8,08
Mo IL-1a (53)	FucmAdMSCs 0h replicate 1	26,00	3,50	1,41	5,44	2,12	2,12
Mo IL-1a (53)	FucmAdMSCs 0h replicate 2	26,00	3,50	1,41	5,44	2,12	2,12
Mo IL-1a (53)	FucmAdMSCs 0h replicate 3	24,50	2,00	0,71	2,89	1,57	1,57
Mo IL-1a (53)	FucmAdMSCs 4h replicate 1	34,00	11,50	0,00	0,00	4,57	4,57
Mo IL-1a (53)	FucmAdMSCs 4h replicate 2	31,50	9,00	0,71	2,24	3,86	3,86
Mo IL-1a (53)	FucmAdMSCs 4h replicate 3	37,80	15,30	0,35	0,94	5,58	5,58
Mo IL-1a (53)	FucmAdMSCs 12h replicate 1	40,00	17,50	0,00	0,00	6,15	6,15
Mo IL-1a (53)	FucmAdMSCs 12h replicate 2	43,00	20,50	1,41	3,29	6,90	6,90
Mo IL-1a (53)	FucmAdMSCs 12h replicate 3	44,00	21,50	1,41	3,21	7,14	7,14
Mo IL-1a (53)	FucmAdMSCs 24h replicate 1	33,50	11,00	2,12	6,33	4,43	4,43
Mo IL-1a (53)	FucmAdMSCs 24h replicate 2	40,00	17,50	2,83	7,07	6,15	6,15
Mo IL-1a (53)	FucmAdMSCs 24h replicate 3	39,50	17,00	0,71	1,79	6,03	6,03
Mo IL-1a (53)	FucmAdMSCs 48h replicate 1	38,00	15,50	5,66	14,89	5,64	5,64
Mo IL-1a (53)	FucmAdMSCs 48h replicate 2	48,00	25,50	0,00	0,00	8,08	8,08
Mo IL-1a (53)	FucmAdMSCs 48h replicate 3	47,80	25,30	2,47	5,18	8,02	8,02
Mo IL-1a (53)	FucmAdMSCs 72h replicate 1	45,50	23,00	3,54	7,77	7,50	7,50
Mo IL-1a (53)	FucmAdMSCs 72h replicate 2	51,00	28,50	0,00	0,00	8,77	8,77
Mo IL-1a (53)	FucmAdMSCs 72h replicate 3	50,80	28,30	1,06	2,09	8,71	8,71
Mo IL-1a (53)	R150	79,00	56,50	4,36	5,52	58,32	58,32
Mo IL-1a (53)	R151	88,80	66,30	5,35	6,02	65,80	65,80
Mo IL-1b (19)	Reagent Name: Background0	9,00	9,00	0,00	0,00		
Mo IL-1b (19)	UmAdMSCs 0h replicate 1	9,00	0,00	0,00	0,00	OOR <	*0.30
Mo IL-1b (19)	UmAdMSCs 0h replicate 2	9,50	0,50	0,71	7,44	OOR <	*0.81
Mo IL-1b (19)	UmAdMSCs 0h replicate 3	9,50	0,50	0,71	7,44	OOR <	*0.81
Mo IL-1b (19)	UmAdMSCs 4h replicate 1	14,00	5,00	0,00	0,00	4,20	4,20
Mo IL-1b (19)	UmAdMSCs 4h replicate 2	16,50	7,50	0,71	4,29	5,81	5,81
Mo IL-1b (19)	UmAdMSCs 4h replicate 3	18,50	9,50	0,71	3,82	7,02	7,02
Mo IL-1b (19)	UmAdMSCs 12h replicate 1	20,50	11,50	0,71	3,45	8,20	8,20
Mo IL-1b (19)	UmAdMSCs 12h replicate 2	60,50	51,50	44,55	73,63	27,99	27,99
Mo IL-1b (19)	UmAdMSCs 12h replicate 3	29,00	20,00	1,41	4,88	12,87	12,87
Mo IL-1b (19)	UmAdMSCs 24h replicate 1	23,80	14,80	0,35	1,49	10,04	10,04
Mo IL-1b (19)	UmAdMSCs 24h replicate 2	21,00	12,00	1,41	6,73	8,49	8,49
Mo IL-1b (19)	UmAdMSCs 24h replicate 3	25,50	16,50	2,12	8,32	11,00	11,00
Mo IL-1b (19)	UmAdMSCs 48h replicate 1	34,50	25,50	2,12	6,15	15,70	15,70
Mo IL-1b (19)	UmAdMSCs 48h replicate 2	28,50	19,50	0,71	2,48	12,61	12,61
Mo IL-1b (19)	UmAdMSCs 48h replicate 3	31,00	22,00	1,41	4,56	13,91	13,91
Mo IL-1b (19)	UmAdMSCs 72h replicate 1	47,00	38,00	2,83	6,02	21,79	21,79
Mo IL-1b (19)	UmAdMSCs 72h replicate 2	48,50	39,50	0,71	1,46	22,50	22,50
Mo IL-1b (19)	UmAdMSCs 72h replicate 3	41,00	32,00	1,41	3,45	18,92	18,92
Mo IL-1b (19)	FucmAdMSCs 0h replicate 1	9,50	0,50	0,00	0,00	OOR <	*0.81
Mo IL-1b (19)	FucmAdMSCs 0h replicate 2	9,00	0,00	0,00	0,00	OOR <	*0.30
Mo IL-1b (19)	FucmAdMSCs 0h replicate 3	9,00	0,00	0,00	0,00	OOR <	*0.30
Mo IL-1b (19)	FucmAdMSCs 4h replicate 1	19,00	10,00	0,00	0,00	7,32	7,32
Mo IL-1b (19)	FucmAdMSCs 4h replicate 2	18,50	9,50	0,71	3,82	7,02	7,02
Mo IL-1b (19)	FucmAdMSCs 4h replicate 3	22,00	13,00	1,41	6,43	9,06	9,06
Mo IL-1b (19)	FucmAdMSCs 12h replicate 1	28,80	19,80	0,35	1,23	12,74	12,74
Mo IL-1b (19)	FucmAdMSCs 12h replicate 2	32,80	23,80	0,35	1,08	14,82	14,82
Mo IL-1b (19)	FucmAdMSCs 12h replicate 3	32,00	23,00	1,41	4,42	14,43	14,43
Mo IL-1b (19)	FucmAdMSCs 24h replicate 1	15,50	6,50	0,71	4,56	5,18	5,18
Mo IL-1b (19)	FucmAdMSCs 24h replicate 2	23,50	14,50	0,71	3,01	9,90	9,90

Mo IL-1b (19)	FucmAdMSCs 24h replicate 3	27,00	18,00	0,00	0,00	11,81	11,81
Mo IL-1b (19)	FucmAdMSCs 48h replicate 1	19,50	10,50	2,12	10,88	7,62	7,62
Mo IL-1b (19)	FucmAdMSCs 48h replicate 2	36,30	27,30	0,35	0,98	16,58	16,58
Mo IL-1b (19)	FucmAdMSCs 48h replicate 3	36,00	27,00	1,41	3,93	16,46	16,46
Mo IL-1b (19)	FucmAdMSCs 72h replicate 1	33,00	24,00	1,41	4,29	14,94	14,94
Mo IL-1b (19)	FucmAdMSCs 72h replicate 2	45,50	36,50	0,71	1,55	21,08	21,08
Mo IL-1b (19)	FucmAdMSCs 72h replicate 3	44,00	35,00	0,00	0,00	20,37	20,37
Mo IL-1b (19)	R150	58,30	49,30	0,58	0,99	108,07	108,07
Mo IL-1b (19)	R151	63,80	54,80	5,92	9,28	117,90	117,90
Mo IL-2 (36)	Reagent Name: Background0	16,00	16,00	1,41	8,84		
Mo IL-2 (36)	UmAdMSCs 0h replicate 1	16,50	0,50	0,71	4,29	OOR <	OOR <
Mo IL-2 (36)	UmAdMSCs 0h replicate 2	15,00	-1,00	1,41	9,43	OOR <	OOR <
Mo IL-2 (36)	UmAdMSCs 0h replicate 3	16,00	0,00	0,00	0,00	OOR <	OOR <
Mo IL-2 (36)	UmAdMSCs 4h replicate 1	18,80	2,80	0,35	1,89	OOR <	*2.19
Mo IL-2 (36)	UmAdMSCs 4h replicate 2	20,50	4,50	0,71	3,45	3,59	3,59
Mo IL-2 (36)	UmAdMSCs 4h replicate 3	21,00	5,00	0,00	0,00	3,96	3,96
Mo IL-2 (36)	UmAdMSCs 12h replicate 1	21,00	5,00	1,41	6,73	3,96	3,96
Mo IL-2 (36)	UmAdMSCs 12h replicate 2	66,00	50,00	56,57	85,71	26,25	26,25
Mo IL-2 (36)	UmAdMSCs 12h replicate 3	27,50	11,50	2,12	7,71	8,13	8,13
Mo IL-2 (36)	UmAdMSCs 24h replicate 1	24,50	8,50	0,71	2,89	6,31	6,31
Mo IL-2 (36)	UmAdMSCs 24h replicate 2	21,00	5,00	0,00	0,00	3,96	3,96
Mo IL-2 (36)	UmAdMSCs 24h replicate 3	27,00	11,00	0,00	0,00	7,83	7,83
Mo IL-2 (36)	UmAdMSCs 48h replicate 1	31,00	15,00	0,00	0,00	10,10	10,10
Mo IL-2 (36)	UmAdMSCs 48h replicate 2	24,50	8,50	2,12	8,66	6,31	6,31
Mo IL-2 (36)	UmAdMSCs 48h replicate 3	27,00	11,00	0,00	0,00	7,83	7,83
Mo IL-2 (36)	UmAdMSCs 72h replicate 1	35,50	19,50	2,12	5,98	12,48	12,48
Mo IL-2 (36)	UmAdMSCs 72h replicate 2	38,30	22,30	0,35	0,92	13,87	13,87
Mo IL-2 (36)	UmAdMSCs 72h replicate 3	33,50	17,50	0,71	2,11	11,44	11,44
Mo IL-2 (36)	FucmAdMSCs 0h replicate 1	15,50	-0,50	0,71	4,56	OOR <	OOR <
Mo IL-2 (36)	FucmAdMSCs 0h replicate 2	16,00	0,00	0,00	0,00	OOR <	OOR <
Mo IL-2 (36)	FucmAdMSCs 0h replicate 3	15,00	-1,00	0,00	0,00	OOR <	OOR <
Mo IL-2 (36)	FucmAdMSCs 4h replicate 1	21,00	5,00	0,00	0,00	3,96	3,96
Mo IL-2 (36)	FucmAdMSCs 4h replicate 2	19,80	3,80	0,35	1,79	3,01	3,01
Mo IL-2 (36)	FucmAdMSCs 4h replicate 3	24,50	8,50	0,71	2,89	6,31	6,31
Mo IL-2 (36)	FucmAdMSCs 12h replicate 1	26,00	10,00	0,00	0,00	7,24	7,24
Mo IL-2 (36)	FucmAdMSCs 12h replicate 2	29,00	13,00	0,00	0,00	8,99	8,99
Mo IL-2 (36)	FucmAdMSCs 12h replicate 3	28,50	12,50	0,71	2,48	8,71	8,71
Mo IL-2 (36)	FucmAdMSCs 24h replicate 1	20,00	4,00	1,41	7,07	3,21	3,21
Mo IL-2 (36)	FucmAdMSCs 24h replicate 2	23,50	7,50	0,71	3,01	5,67	5,67
Mo IL-2 (36)	FucmAdMSCs 24h replicate 3	24,00	8,00	1,41	5,89	5,99	5,99
Mo IL-2 (36)	FucmAdMSCs 48h replicate 1	21,00	5,00	1,41	6,73	3,96	3,96
Mo IL-2 (36)	FucmAdMSCs 48h replicate 2	31,00	15,00	0,00	0,00	10,10	10,10
Mo IL-2 (36)	FucmAdMSCs 48h replicate 3	29,00	13,00	0,00	0,00	8,99	8,99
Mo IL-2 (36)	FucmAdMSCs 72h replicate 1	27,50	11,50	0,71	2,57	8,13	8,13
Mo IL-2 (36)	FucmAdMSCs 72h replicate 2	35,50	19,50	0,71	1,99	12,48	12,48
Mo IL-2 (36)	FucmAdMSCs 72h replicate 3	35,50	19,50	0,71	1,99	12,48	12,48
Mo IL-2 (36)	R150	72,50	56,50	1,32	1,82	115,56	115,56
Mo IL-2 (36)	R151	89,00	73,00	6,56	7,37	141,16	141,16
Mo IL-3 (18)	Reagent Name: Background0	15,80	15,80	0,35	2,24		
Mo IL-3 (18)	UmAdMSCs 0h replicate 1	18,50	2,80	0,71	3,82	1,33	1,33
Mo IL-3 (18)	UmAdMSCs 0h replicate 2	17,00	1,30	1,41	8,32	OOR <	*0.92
Mo IL-3 (18)	UmAdMSCs 0h replicate 3	17,50	1,80	0,71	4,04	1,06	1,06
Mo IL-3 (18)	UmAdMSCs 4h replicate 1	19,00	3,30	0,00	0,00	1,46	1,46
Mo IL-3 (18)	UmAdMSCs 4h replicate 2	21,00	5,30	0,00	0,00	1,98	1,98
Mo IL-3 (18)	UmAdMSCs 4h replicate 3	21,00	5,30	0,00	0,00	1,98	1,98
Mo IL-3 (18)	UmAdMSCs 12h replicate 1	22,00	6,30	1,41	6,43	2,23	2,23
Mo IL-3 (18)	UmAdMSCs 12h replicate 2	60,50	44,80	45,96	75,97	10,59	10,59
Mo IL-3 (18)	UmAdMSCs 12h replicate 3	29,50	13,80	0,71	2,40	4,00	4,00
Mo IL-3 (18)	UmAdMSCs 24h replicate 1	25,50	9,80	2,12	8,32	3,07	3,07
Mo IL-3 (18)	UmAdMSCs 24h replicate 2	24,00	8,30	1,41	5,89	2,71	2,71
Mo IL-3 (18)	UmAdMSCs 24h replicate 3	25,80	10,00	1,77	6,87	3,13	3,13
Mo IL-3 (18)	UmAdMSCs 48h replicate 1	32,00	16,30	1,41	4,42	4,56	4,56
Mo IL-3 (18)	UmAdMSCs 48h replicate 2	27,50	11,80	2,12	7,71	3,54	3,54
Mo IL-3 (18)	UmAdMSCs 48h replicate 3	29,50	13,80	0,71	2,40	4,00	4,00
Mo IL-3 (18)	UmAdMSCs 72h replicate 1	39,50	23,80	0,71	1,79	6,21	6,21
Mo IL-3 (18)	UmAdMSCs 72h replicate 2	42,50	26,80	2,12	4,99	6,85	6,85
Mo IL-3 (18)	UmAdMSCs 72h replicate 3	36,50	20,80	0,71	1,94	5,56	5,56
Mo IL-3 (18)	FucmAdMSCs 0h replicate 1	17,00	1,30	0,00	0,00	OOR <	*0.92
Mo IL-3 (18)	FucmAdMSCs 0h replicate 2	17,00	1,30	0,00	0,00	OOR <	*0.92
Mo IL-3 (18)	FucmAdMSCs 0h replicate 3	16,50	0,80	0,71	4,29	OOR <	*0.78

Mo IL-3 (18)	FucmAdMSCs 4h replicate 1	22,50	6,80	0,71	3,14	2,35	2,35
Mo IL-3 (18)	FucmAdMSCs 4h replicate 2	20,80	5,00	1,06	5,11	1,91	1,91
Mo IL-3 (18)	FucmAdMSCs 4h replicate 3	25,50	9,80	0,71	2,77	3,07	3,07
Mo IL-3 (18)	FucmAdMSCs 12h replicate 1	27,30	11,50	0,35	1,30	3,48	3,48
Mo IL-3 (18)	FucmAdMSCs 12h replicate 2	30,00	14,30	1,41	4,71	4,11	4,11
Mo IL-3 (18)	FucmAdMSCs 12h replicate 3	30,00	14,30	0,00	0,00	4,11	4,11
Mo IL-3 (18)	FucmAdMSCs 24h replicate 1	21,50	5,80	0,71	3,29	2,10	2,10
Mo IL-3 (18)	FucmAdMSCs 24h replicate 2	25,00	9,30	1,41	5,66	2,95	2,95
Mo IL-3 (18)	FucmAdMSCs 24h replicate 3	28,50	12,80	0,71	2,48	3,77	3,77
Mo IL-3 (18)	FucmAdMSCs 48h replicate 1	24,00	8,30	2,83	11,79	2,71	2,71
Mo IL-3 (18)	FucmAdMSCs 48h replicate 2	34,50	18,80	0,71	2,05	5,12	5,12
Mo IL-3 (18)	FucmAdMSCs 48h replicate 3	34,00	18,30	1,41	4,16	5,01	5,01
Mo IL-3 (18)	FucmAdMSCs 72h replicate 1	30,50	14,80	0,71	2,32	4,22	4,22
Mo IL-3 (18)	FucmAdMSCs 72h replicate 2	38,50	22,80	0,71	1,84	5,99	5,99
Mo IL-3 (18)	FucmAdMSCs 72h replicate 3	38,00	22,30	1,41	3,72	5,88	5,88
Mo IL-3 (18)	R150	48,30	32,60	1,53	3,16	32,33	32,33
Mo IL-3 (18)	R151	51,20	35,40	3,33	6,51	34,69	34,69
Mo IL-4 (39)	Reagent Name: Background0	34,50	34,50	0,71	2,05		
Mo IL-4 (39)	UmAdMSCs 0h replicate 1	41,50	7,00	0,71	1,70	3,55	3,55
Mo IL-4 (39)	UmAdMSCs 0h replicate 2	41,00	6,50	4,24	10,35	3,41	3,41
Mo IL-4 (39)	UmAdMSCs 0h replicate 3	42,00	7,50	0,00	0,00	3,70	3,70
Mo IL-4 (39)	UmAdMSCs 4h replicate 1	44,00	9,50	7,07	16,07	4,26	4,26
Mo IL-4 (39)	UmAdMSCs 4h replicate 2	39,50	5,00	0,71	1,79	OOR <	*2.97
Mo IL-4 (39)	UmAdMSCs 4h replicate 3	48,80	14,30	11,67	23,93	5,56	5,56
Mo IL-4 (39)	UmAdMSCs 12h replicate 1	39,00	4,50	4,24	10,88	OOR <	*2.82
Mo IL-4 (39)	UmAdMSCs 12h replicate 2	108,50	74,00	74,25	68,43	20,00	20,00
Mo IL-4 (39)	UmAdMSCs 12h replicate 3	45,50	11,00	0,71	1,55	4,68	4,68
Mo IL-4 (39)	UmAdMSCs 24h replicate 1	46,00	11,50	5,66	12,30	4,82	4,82
Mo IL-4 (39)	UmAdMSCs 24h replicate 2	45,50	11,00	3,54	7,77	4,68	4,68
Mo IL-4 (39)	UmAdMSCs 24h replicate 3	46,00	11,50	1,41	3,07	4,82	4,82
Mo IL-4 (39)	UmAdMSCs 48h replicate 1	48,50	14,00	9,19	18,95	5,50	5,50
Mo IL-4 (39)	UmAdMSCs 48h replicate 2	42,50	8,00	0,71	1,66	3,84	3,84
Mo IL-4 (39)	UmAdMSCs 48h replicate 3	39,50	5,00	0,71	1,79	OOR <	*2.97
Mo IL-4 (39)	UmAdMSCs 72h replicate 1	49,50	15,00	7,78	15,71	5,77	5,77
Mo IL-4 (39)	UmAdMSCs 72h replicate 2	48,50	14,00	0,71	1,46	5,50	5,50
Mo IL-4 (39)	UmAdMSCs 72h replicate 3	46,00	11,50	4,24	9,22	4,82	4,82
Mo IL-4 (39)	FucmAdMSCs 0h replicate 1	43,00	8,50	2,83	6,58	3,98	3,98
Mo IL-4 (39)	FucmAdMSCs 0h replicate 2	36,80	2,30	0,35	0,96	OOR <	*2.14
Mo IL-4 (39)	FucmAdMSCs 0h replicate 3	36,00	1,50	0,00	0,00	OOR <	*1.90
Mo IL-4 (39)	FucmAdMSCs 4h replicate 1	39,00	4,50	0,00	0,00	OOR <	*2.82
Mo IL-4 (39)	FucmAdMSCs 4h replicate 2	35,00	0,50	0,00	0,00	OOR <	*1.58
Mo IL-4 (39)	FucmAdMSCs 4h replicate 3	38,50	4,00	0,71	1,84	OOR <	*2.67
Mo IL-4 (39)	FucmAdMSCs 12h replicate 1	40,00	5,50	1,41	3,54	OOR <	*3.12
Mo IL-4 (39)	FucmAdMSCs 12h replicate 2	41,50	7,00	1,41	3,41	3,55	3,55
Mo IL-4 (39)	FucmAdMSCs 12h replicate 3	44,50	10,00	2,12	4,77	4,40	4,40
Mo IL-4 (39)	FucmAdMSCs 24h replicate 1	35,00	0,50	0,00	0,00	OOR <	*1.58
Mo IL-4 (39)	FucmAdMSCs 24h replicate 2	40,50	6,00	2,12	5,24	OOR <	*3.26
Mo IL-4 (39)	FucmAdMSCs 24h replicate 3	39,00	4,50	2,83	7,25	OOR <	*2.82
Mo IL-4 (39)	FucmAdMSCs 48h replicate 1	37,00	2,50	2,83	7,64	OOR <	*2.21
Mo IL-4 (39)	FucmAdMSCs 48h replicate 2	41,00	6,50	1,41	3,45	3,41	3,41
Mo IL-4 (39)	FucmAdMSCs 48h replicate 3	43,80	9,30	1,06	2,42	4,19	4,19
Mo IL-4 (39)	FucmAdMSCs 72h replicate 1	44,50	10,00	2,12	4,77	4,40	4,40
Mo IL-4 (39)	FucmAdMSCs 72h replicate 2	50,50	16,00	6,36	12,60	6,03	6,03
Mo IL-4 (39)	FucmAdMSCs 72h replicate 3	45,50	11,00	4,95	10,88	4,68	4,68
Mo IL-4 (39)	R150	124,30	89,80	5,86	4,71	94,19	94,19
Mo IL-4 (39)	R151	121,00	86,50	11,27	9,31	91,23	91,23
Mo IL-5 (52)	Reagent Name: Background0	9,50	9,50	0,71	7,44		
Mo IL-5 (52)	UmAdMSCs 0h replicate 1	11,00	1,50	0,00	0,00	OOR <	OOR <
Mo IL-5 (52)	UmAdMSCs 0h replicate 2	11,00	1,50	1,41	12,86	OOR <	OOR <
Mo IL-5 (52)	UmAdMSCs 0h replicate 3	10,00	0,50	0,00	0,00	OOR <	OOR <
Mo IL-5 (52)	UmAdMSCs 4h replicate 1	10,80	1,30	1,06	9,87	OOR <	OOR <
Mo IL-5 (52)	UmAdMSCs 4h replicate 2	12,50	3,00	0,71	5,66	OOR <	*1.23
Mo IL-5 (52)	UmAdMSCs 4h replicate 3	12,50	3,00	0,71	5,66	OOR <	*1.23
Mo IL-5 (52)	UmAdMSCs 12h replicate 1	13,00	3,50	0,00	0,00	OOR <	*1.89
Mo IL-5 (52)	UmAdMSCs 12h replicate 2	50,00	40,50	50,91	101,82	24,26	24,26
Mo IL-5 (52)	UmAdMSCs 12h replicate 3	16,00	6,50	0,00	0,00	OOR <	*4.87
Mo IL-5 (52)	UmAdMSCs 24h replicate 1	15,30	5,80	0,35	2,32	OOR <	*4.21
Mo IL-5 (52)	UmAdMSCs 24h replicate 2	13,50	4,00	0,71	5,24	OOR <	*2.47
Mo IL-5 (52)	UmAdMSCs 24h replicate 3	16,00	6,50	0,00	0,00	OOR <	*4.87
Mo IL-5 (52)	UmAdMSCs 48h replicate 1	17,00	7,50	0,00	0,00	5,69	5,69

Mo IL-5 (52)	UmAdMSCs 48h replicate 2	16,00	6,50	1,41	8,84	OOR <	*4.87
Mo IL-5 (52)	UmAdMSCs 48h replicate 3	16,50	7,00	0,71	4,29	OOR <	*5.29
Mo IL-5 (52)	UmAdMSCs 72h replicate 1	21,00	11,50	1,41	6,73	8,64	8,64
Mo IL-5 (52)	UmAdMSCs 72h replicate 2	23,00	13,50	0,00	0,00	9,97	9,97
Mo IL-5 (52)	UmAdMSCs 72h replicate 3	20,30	10,80	0,35	1,75	8,12	8,12
Mo IL-5 (52)	FucmAdMSCs 0h replicate 1	10,50	1,00	0,71	6,73	OOR <	OOR <
Mo IL-5 (52)	FucmAdMSCs 0h replicate 2	11,00	1,50	0,00	0,00	OOR <	OOR <
Mo IL-5 (52)	FucmAdMSCs 0h replicate 3	10,00	0,50	0,00	0,00	OOR <	OOR <
Mo IL-5 (52)	FucmAdMSCs 4h replicate 1	12,50	3,00	0,71	5,66	OOR <	*1.23
Mo IL-5 (52)	FucmAdMSCs 4h replicate 2	12,50	3,00	0,71	5,66	OOR <	*1.23
Mo IL-5 (52)	FucmAdMSCs 4h replicate 3	14,00	4,50	0,00	0,00	OOR <	*3.01
Mo IL-5 (52)	FucmAdMSCs 12h replicate 1	16,00	6,50	0,00	0,00	OOR <	*4.87
Mo IL-5 (52)	FucmAdMSCs 12h replicate 2	17,00	7,50	1,41	8,32	5,69	5,69
Mo IL-5 (52)	FucmAdMSCs 12h replicate 3	17,00	7,50	0,00	0,00	5,69	5,69
Mo IL-5 (52)	FucmAdMSCs 24h replicate 1	12,50	3,00	0,71	5,66	OOR <	*1.23
Mo IL-5 (52)	FucmAdMSCs 24h replicate 2	14,50	5,00	0,71	4,88	OOR <	*3.51
Mo IL-5 (52)	FucmAdMSCs 24h replicate 3	15,50	6,00	0,71	4,56	OOR <	*4.43
Mo IL-5 (52)	FucmAdMSCs 48h replicate 1	14,00	4,50	1,41	10,10	OOR <	*3.01
Mo IL-5 (52)	FucmAdMSCs 48h replicate 2	18,00	8,50	1,41	7,86	6,48	6,48
Mo IL-5 (52)	FucmAdMSCs 48h replicate 3	19,00	9,50	1,41	7,44	7,23	7,23
Mo IL-5 (52)	FucmAdMSCs 72h replicate 1	17,00	7,50	0,00	0,00	5,69	5,69
Mo IL-5 (52)	FucmAdMSCs 72h replicate 2	19,50	10,00	0,71	3,63	7,59	7,59
Mo IL-5 (52)	FucmAdMSCs 72h replicate 3	21,00	11,50	0,00	0,00	8,64	8,64
Mo IL-5 (52)	R150	52,70	43,20	3,79	7,19	101,88	101,88
Mo IL-5 (52)	R151	66,50	57,00	6,50	9,77	125,78	125,78
Mo IL-6 (38)	Reagent Name: Background0	23,00	23,00	1,41	6,15		
Mo IL-6 (38)	UmAdMSCs 0h replicate 1	41,80	18,80	13,79	33,03	2,29	2,29
Mo IL-6 (38)	UmAdMSCs 0h replicate 2	28,50	5,50	0,71	2,48	0,99	0,99
Mo IL-6 (38)	UmAdMSCs 0h replicate 3	27,50	4,50	0,71	2,57	0,88	0,88
Mo IL-6 (38)	UmAdMSCs 4h replicate 1	1954,30	1931,30	8,13	0,42	147,57	147,57
Mo IL-6 (38)	UmAdMSCs 4h replicate 2	3090,30	3067,30	12,37	0,40	237,07	237,07
Mo IL-6 (38)	UmAdMSCs 4h replicate 3	3489,50	3466,50	197,28	5,65	269,93	269,93
Mo IL-6 (38)	UmAdMSCs 12h replicate 1	4051,30	4028,30	54,09	1,34	317,56	317,56
Mo IL-6 (38)	UmAdMSCs 12h replicate 2	5735,30	5712,30	157,33	2,74	471,49	471,49
Mo IL-6 (38)	UmAdMSCs 12h replicate 3	6299,50	6276,50	161,93	2,57	527,26	527,26
Mo IL-6 (38)	UmAdMSCs 24h replicate 1	4521,00	4498,00	175,36	3,88	358,75	358,75
Mo IL-6 (38)	UmAdMSCs 24h replicate 2	3678,00	3655,00	260,22	7,07	285,72	285,72
Mo IL-6 (38)	UmAdMSCs 24h replicate 3	4693,30	4670,30	166,52	3,55	374,17	374,17
Mo IL-6 (38)	UmAdMSCs 48h replicate 1	7277,80	7254,80	208,24	2,86	629,60	629,60
Mo IL-6 (38)	UmAdMSCs 48h replicate 2	5989,30	5966,30	184,91	3,09	496,32	496,32
Mo IL-6 (38)	UmAdMSCs 48h replicate 3	7310,00	7287,00	59,40	0,81	633,10	633,10
Mo IL-6 (38)	UmAdMSCs 72h replicate 1	9443,80	9420,80	22,27	0,24	885,55	885,55
Mo IL-6 (38)	UmAdMSCs 72h replicate 2	10296,50	10273,50	73,54	0,71	999,25	999,25
Mo IL-6 (38)	UmAdMSCs 72h replicate 3	8474,30	8451,30	0,35	0,00	765,56	765,56
Mo IL-6 (38)	FucmAdMSCs 0h replicate 1	28,00	5,00	0,00	0,00	0,93	0,93
Mo IL-6 (38)	FucmAdMSCs 0h replicate 2	38,50	15,50	14,85	38,57	1,98	1,98
Mo IL-6 (38)	FucmAdMSCs 0h replicate 3	26,30	3,30	0,35	1,35	OOR <	*0.75
Mo IL-6 (38)	FucmAdMSCs 4h replicate 1	3571,80	3548,80	100,76	2,82	276,80	276,80
Mo IL-6 (38)	FucmAdMSCs 4h replicate 2	4114,30	4091,30	199,76	4,86	323,01	323,01
Mo IL-6 (38)	FucmAdMSCs 4h replicate 3	4487,80	4464,80	153,80	3,43	355,79	355,79
Mo IL-6 (38)	FucmAdMSCs 12h replicate 1	6461,50	6438,50	41,72	0,65	543,70	543,70
Mo IL-6 (38)	FucmAdMSCs 12h replicate 2	7370,30	7347,30	5,30	0,07	639,67	639,67
Mo IL-6 (38)	FucmAdMSCs 12h replicate 3	7050,00	7027,00	56,57	0,80	605,10	605,10
Mo IL-6 (38)	FucmAdMSCs 24h replicate 1	2547,00	2524,00	151,32	5,94	193,59	193,59
Mo IL-6 (38)	FucmAdMSCs 24h replicate 2	4747,30	4724,30	216,02	4,55	379,05	379,05
Mo IL-6 (38)	FucmAdMSCs 24h replicate 3	6063,00	6040,00	130,11	2,15	503,61	503,61
Mo IL-6 (38)	FucmAdMSCs 48h replicate 1	4542,00	4519,00	154,86	3,41	360,62	360,62
Mo IL-6 (38)	FucmAdMSCs 48h replicate 2	7458,00	7435,00	75,66	1,01	649,29	649,29
Mo IL-6 (38)	FucmAdMSCs 48h replicate 3	7903,80	7880,80	479,77	6,07	699,16	699,16
Mo IL-6 (38)	FucmAdMSCs 72h replicate 1	6718,50	6695,50	147,79	2,20	570,19	570,19
Mo IL-6 (38)	FucmAdMSCs 72h replicate 2	10275,00	10252,00	171,12	1,67	996,28	996,28
Mo IL-6 (38)	FucmAdMSCs 72h replicate 3	10154,50	10131,50	654,07	6,44	979,75	979,75
Mo IL-6 (38)	R150	106,50	83,50	8,50	7,98	31,91	31,91
Mo IL-6 (38)	R151	132,80	109,80	16,02	12,06	40,65	40,65
Mo IL-9 (33)	Reagent Name: Background0	78,00	78,00	0,00	0,00		
Mo IL-9 (33)	UmAdMSCs 0h replicate 1	83,00	5,00	0,00	0,00	11,79	11,79
Mo IL-9 (33)	UmAdMSCs 0h replicate 2	80,00	2,00	5,66	7,07	8,45	8,45
Mo IL-9 (33)	UmAdMSCs 0h replicate 3	79,50	1,50	0,71	0,89	7,86	7,86
Mo IL-9 (33)	UmAdMSCs 4h replicate 1	81,50	3,50	3,54	4,34	10,16	10,16
Mo IL-9 (33)	UmAdMSCs 4h replicate 2	83,50	5,50	0,71	0,85	12,32	12,32

Mo IL-9 (33)	UmAdMSCs 4h replicate 3	82,50	4,50	2,12	2,57	11,26	11,26
Mo IL-9 (33)	UmAdMSCs 12h replicate 1	77,50	-0,50	7,78	10,04	OOR <	*5.33
Mo IL-9 (33)	UmAdMSCs 12h replicate 2	119,30	41,30	39,24	32,91	42,48	42,48
Mo IL-9 (33)	UmAdMSCs 12h replicate 3	95,00	17,00	1,41	1,49	23,23	23,23
Mo IL-9 (33)	UmAdMSCs 24h replicate 1	89,00	11,00	1,41	1,59	17,79	17,79
Mo IL-9 (33)	UmAdMSCs 24h replicate 2	88,50	10,50	0,71	0,80	17,31	17,31
Mo IL-9 (33)	UmAdMSCs 24h replicate 3	91,00	13,00	5,66	6,22	19,65	19,65
Mo IL-9 (33)	UmAdMSCs 48h replicate 1	99,80	21,80	2,47	2,48	27,29	27,29
Mo IL-9 (33)	UmAdMSCs 48h replicate 2	98,00	20,00	4,24	4,33	25,82	25,82
Mo IL-9 (33)	UmAdMSCs 48h replicate 3	92,80	14,80	1,06	1,14	21,24	21,24
Mo IL-9 (33)	UmAdMSCs 72h replicate 1	110,50	32,50	6,36	5,76	35,90	35,90
Mo IL-9 (33)	UmAdMSCs 72h replicate 2	116,00	38,00	4,24	3,66	40,07	40,07
Mo IL-9 (33)	UmAdMSCs 72h replicate 3	107,30	29,30	0,35	0,33	33,37	33,37
Mo IL-9 (33)	FucmAdMSCs 0h replicate 1	79,50	1,50	2,12	2,67	7,86	7,86
Mo IL-9 (33)	FucmAdMSCs 0h replicate 2	78,00	0,00	0,00	0,00	OOR <	*5.99
Mo IL-9 (33)	FucmAdMSCs 0h replicate 3	74,50	-3,50	0,71	0,95	OOR <	*0.31
Mo IL-9 (33)	FucmAdMSCs 4h replicate 1	86,30	8,30	0,35	0,41	15,13	15,13
Mo IL-9 (33)	FucmAdMSCs 4h replicate 2	75,00	-3,00	1,41	1,89	OOR <	*1.46
Mo IL-9 (33)	FucmAdMSCs 4h replicate 3	87,00	9,00	1,41	1,63	15,87	15,87
Mo IL-9 (33)	FucmAdMSCs 12h replicate 1	90,00	12,00	0,00	0,00	18,73	18,73
Mo IL-9 (33)	FucmAdMSCs 12h replicate 2	90,00	12,00	0,00	0,00	18,73	18,73
Mo IL-9 (33)	FucmAdMSCs 12h replicate 3	95,50	17,50	2,12	2,22	23,67	23,67
Mo IL-9 (33)	FucmAdMSCs 24h replicate 1	82,80	4,80	2,47	2,99	11,53	11,53
Mo IL-9 (33)	FucmAdMSCs 24h replicate 2	90,00	12,00	7,07	7,86	18,73	18,73
Mo IL-9 (33)	FucmAdMSCs 24h replicate 3	88,00	10,00	7,07	8,04	16,83	16,83
Mo IL-9 (33)	FucmAdMSCs 48h replicate 1	85,00	7,00	9,90	11,65	13,87	13,87
Mo IL-9 (33)	FucmAdMSCs 48h replicate 2	105,30	27,30	0,35	0,34	31,78	31,78
Mo IL-9 (33)	FucmAdMSCs 48h replicate 3	104,50	26,50	6,36	6,09	31,18	31,18
Mo IL-9 (33)	FucmAdMSCs 72h replicate 1	100,00	22,00	2,83	2,83	27,50	27,50
Mo IL-9 (33)	FucmAdMSCs 72h replicate 2	109,80	31,80	4,60	4,19	35,32	35,32
Mo IL-9 (33)	FucmAdMSCs 72h replicate 3	113,50	35,50	0,71	0,62	38,19	38,19
Mo IL-9 (33)	R150	117,00	39,00	2,65	2,26	163,28	163,28
Mo IL-9 (33)	R151	125,70	47,70	5,51	4,38	188,49	188,49
Mo IL-10 (56)	Reagent Name: Background0	21,00	21,00	0,00	0,00		
Mo IL-10 (56)	UmAdMSCs 0h replicate 1	25,00	4,00	1,41	5,66	6,69	6,69
Mo IL-10 (56)	UmAdMSCs 0h replicate 2	24,00	3,00	1,41	5,89	5,84	5,84
Mo IL-10 (56)	UmAdMSCs 0h replicate 3	22,50	1,50	0,71	3,14	4,54	4,54
Mo IL-10 (56)	UmAdMSCs 4h replicate 1	26,00	5,00	0,00	0,00	7,53	7,53
Mo IL-10 (56)	UmAdMSCs 4h replicate 2	24,50	3,50	0,71	2,89	6,26	6,26
Mo IL-10 (56)	UmAdMSCs 4h replicate 3	26,50	5,50	0,71	2,67	7,95	7,95
Mo IL-10 (56)	UmAdMSCs 12h replicate 1	26,00	5,00	0,00	0,00	7,53	7,53
Mo IL-10 (56)	UmAdMSCs 12h replicate 2	70,50	49,50	51,62	73,22	41,30	41,30
Mo IL-10 (56)	UmAdMSCs 12h replicate 3	33,50	12,50	0,71	2,11	13,63	13,63
Mo IL-10 (56)	UmAdMSCs 24h replicate 1	31,50	10,50	2,12	6,73	12,03	12,03
Mo IL-10 (56)	UmAdMSCs 24h replicate 2	29,50	8,50	0,71	2,40	10,42	10,42
Mo IL-10 (56)	UmAdMSCs 24h replicate 3	31,50	10,50	0,71	2,24	12,03	12,03
Mo IL-10 (56)	UmAdMSCs 48h replicate 1	34,50	13,50	0,71	2,05	14,42	14,42
Mo IL-10 (56)	UmAdMSCs 48h replicate 2	33,00	12,00	2,83	8,57	13,23	13,23
Mo IL-10 (56)	UmAdMSCs 48h replicate 3	32,00	11,00	2,83	8,84	12,43	12,43
Mo IL-10 (56)	UmAdMSCs 72h replicate 1	39,80	18,80	5,30	13,34	18,52	18,52
Mo IL-10 (56)	UmAdMSCs 72h replicate 2	43,00	22,00	0,71	1,64	21,01	21,01
Mo IL-10 (56)	UmAdMSCs 72h replicate 3	39,50	18,50	2,12	5,37	18,33	18,33
Mo IL-10 (56)	FucmAdMSCs 0h replicate 1	23,00	2,00	1,41	6,15	4,97	4,97
Mo IL-10 (56)	FucmAdMSCs 0h replicate 2	24,00	3,00	0,00	0,00	5,84	5,84
Mo IL-10 (56)	FucmAdMSCs 0h replicate 3	22,50	1,50	0,71	3,14	4,54	4,54
Mo IL-10 (56)	FucmAdMSCs 4h replicate 1	26,50	5,50	0,71	2,67	7,95	7,95
Mo IL-10 (56)	FucmAdMSCs 4h replicate 2	24,50	3,50	0,71	2,89	6,26	6,26
Mo IL-10 (56)	FucmAdMSCs 4h replicate 3	28,50	7,50	2,12	7,44	9,60	9,60
Mo IL-10 (56)	FucmAdMSCs 12h replicate 1	32,00	11,00	1,41	4,42	12,43	12,43
Mo IL-10 (56)	FucmAdMSCs 12h replicate 2	31,00	10,00	2,83	9,12	11,63	11,63
Mo IL-10 (56)	FucmAdMSCs 12h replicate 3	33,50	12,50	0,71	2,11	13,63	13,63
Mo IL-10 (56)	FucmAdMSCs 24h replicate 1	25,50	4,50	0,71	2,77	7,11	7,11
Mo IL-10 (56)	FucmAdMSCs 24h replicate 2	28,50	7,50	0,71	2,48	9,60	9,60
Mo IL-10 (56)	FucmAdMSCs 24h replicate 3	29,50	8,50	0,71	2,40	10,42	10,42
Mo IL-10 (56)	FucmAdMSCs 48h replicate 1	29,00	8,00	1,41	4,88	10,01	10,01
Mo IL-10 (56)	FucmAdMSCs 48h replicate 2	37,50	16,50	0,71	1,89	16,77	16,77
Mo IL-10 (56)	FucmAdMSCs 48h replicate 3	37,00	16,00	1,41	3,82	16,38	16,38
Mo IL-10 (56)	FucmAdMSCs 72h replicate 1	33,00	12,00	1,41	4,29	13,23	13,23
Mo IL-10 (56)	FucmAdMSCs 72h replicate 2	39,30	18,30	0,35	0,90	18,13	18,13
Mo IL-10 (56)	FucmAdMSCs 72h replicate 3	38,50	17,50	2,12	5,51	17,55	17,55

Mo IL-10 (56)	R150	124,20	103,20	10,10	8,14	314,44	314,44
Mo IL-10 (56)	R151	126,80	105,80	10,73	8,46	321,65	321,65
Mo IL-12(p40) (76)	Reagent Name: Background0	18,00	18,00	0,00	0,00		
Mo IL-12(p40) (76)	UmAdMSCs 0h replicate 1	21,00	3,00	0,00	0,00	2,62	2,62
Mo IL-12(p40) (76)	UmAdMSCs 0h replicate 2	20,50	2,50	2,12	10,35	2,39	2,39
Mo IL-12(p40) (76)	UmAdMSCs 0h replicate 3	20,00	2,00	1,41	7,07	2,17	2,17
Mo IL-12(p40) (76)	UmAdMSCs 4h replicate 1	22,50	4,50	0,71	3,14	3,29	3,29
Mo IL-12(p40) (76)	UmAdMSCs 4h replicate 2	26,00	8,00	2,83	10,88	4,82	4,82
Mo IL-12(p40) (76)	UmAdMSCs 4h replicate 3	26,50	8,50	0,71	2,67	5,04	5,04
Mo IL-12(p40) (76)	UmAdMSCs 12h replicate 1	26,80	8,80	0,35	1,32	5,15	5,15
Mo IL-12(p40) (76)	UmAdMSCs 12h replicate 2	72,50	54,50	54,45	75,10	24,44	24,44
Mo IL-12(p40) (76)	UmAdMSCs 12h replicate 3	36,30	18,30	1,06	2,93	9,24	9,24
Mo IL-12(p40) (76)	UmAdMSCs 24h replicate 1	30,30	12,30	1,77	5,84	6,67	6,67
Mo IL-12(p40) (76)	UmAdMSCs 24h replicate 2	28,00	10,00	0,00	0,00	5,70	5,70
Mo IL-12(p40) (76)	UmAdMSCs 24h replicate 3	32,00	14,00	1,41	4,42	7,42	7,42
Mo IL-12(p40) (76)	UmAdMSCs 48h replicate 1	36,50	18,50	2,12	5,81	9,35	9,35
Mo IL-12(p40) (76)	UmAdMSCs 48h replicate 2	34,50	16,50	0,71	2,05	8,50	8,50
Mo IL-12(p40) (76)	UmAdMSCs 48h replicate 3	36,50	18,50	2,12	5,81	9,35	9,35
Mo IL-12(p40) (76)	UmAdMSCs 72h replicate 1	49,00	31,00	1,41	2,89	14,64	14,64
Mo IL-12(p40) (76)	UmAdMSCs 72h replicate 2	53,50	35,50	2,12	3,97	16,53	16,53
Mo IL-12(p40) (76)	UmAdMSCs 72h replicate 3	46,00	28,00	2,83	6,15	13,38	13,38
Mo IL-12(p40) (76)	F 0h replicate 1	20,50	2,50	0,71	3,45	2,39	2,39
Mo IL-12(p40) (76)	F 0h replicate 2	19,50	1,50	0,71	3,63	1,94	1,94
Mo IL-12(p40) (76)	F 0h replicate 3	19,00	1,00	1,41	7,44	1,72	1,72
Mo IL-12(p40) (76)	F 4h replicate 1	26,50	8,50	0,71	2,67	5,04	5,04
Mo IL-12(p40) (76)	F 4h replicate 2	26,00	8,00	0,00	0,00	4,82	4,82
Mo IL-12(p40) (76)	F 4h replicate 3	30,00	12,00	1,41	4,71	6,56	6,56
Mo IL-12(p40) (76)	F 12h replicate 1	35,00	17,00	0,00	0,00	8,71	8,71
Mo IL-12(p40) (76)	F 12h replicate 2	36,50	18,50	0,71	1,94	9,35	9,35
Mo IL-12(p40) (76)	F 12h replicate 3	37,00	19,00	0,00	0,00	9,56	9,56
Mo IL-12(p40) (76)	F 24h replicate 1	25,50	7,50	0,71	2,77	4,61	4,61
Mo IL-12(p40) (76)	F 24h replicate 2	30,50	12,50	0,71	2,32	6,78	6,78
Mo IL-12(p40) (76)	F 24h replicate 3	33,50	15,50	0,71	2,11	8,07	8,07
Mo IL-12(p40) (76)	F 48h replicate 1	28,80	10,80	1,77	6,15	6,02	6,02
Mo IL-12(p40) (76)	F 48h replicate 2	42,00	24,00	0,00	0,00	11,69	11,69
Mo IL-12(p40) (76)	F 48h replicate 3	43,00	25,00	2,83	6,58	12,11	12,11
Mo IL-12(p40) (76)	F 72h replicate 1	37,50	19,50	2,12	5,66	9,78	9,78
Mo IL-12(p40) (76)	F 72h replicate 2	49,30	31,30	0,35	0,72	14,75	14,75
Mo IL-12(p40) (76)	F 72h replicate 3	46,80	28,80	0,35	0,76	13,69	13,69
Mo IL-12(p40) (76)	R150	395,80	377,80	19,00	4,80	627,48	627,48
Mo IL-12(p40) (76)	R151	525,30	507,30	4,04	0,77	841,81	841,81
Mo IL-12(p70) (78)	Reagent Name: Background0	68,00	68,00	1,41	2,08		
Mo IL-12(p70) (78)	UmAdMSCs 0h replicate 1	80,00	12,00	1,41	1,77	28,27	28,27
Mo IL-12(p70) (78)	UmAdMSCs 0h replicate 2	76,50	8,50	4,95	6,47	22,97	22,97
Mo IL-12(p70) (78)	UmAdMSCs 0h replicate 3	76,00	8,00	1,41	1,86	22,22	22,22
Mo IL-12(p70) (78)	UmAdMSCs 4h replicate 1	98,80	30,80	3,89	3,94	56,92	56,92
Mo IL-12(p70) (78)	UmAdMSCs 4h replicate 2	113,00	45,00	0,00	0,00	78,97	78,97
Mo IL-12(p70) (78)	UmAdMSCs 4h replicate 3	115,50	47,50	4,95	4,29	82,86	82,86
Mo IL-12(p70) (78)	UmAdMSCs 12h replicate 1	111,50	43,50	9,19	8,24	76,64	76,64
Mo IL-12(p70) (78)	UmAdMSCs 12h replicate 2	178,50	110,50	54,45	30,50	182,76	182,76
Mo IL-12(p70) (78)	UmAdMSCs 12h replicate 3	143,50	75,50	0,71	0,49	126,84	126,84
Mo IL-12(p70) (78)	UmAdMSCs 24h replicate 1	126,50	58,50	4,95	3,91	100,05	100,05
Mo IL-12(p70) (78)	UmAdMSCs 24h replicate 2	124,30	56,30	1,06	0,85	96,53	96,53
Mo IL-12(p70) (78)	UmAdMSCs 24h replicate 3	126,00	58,00	2,83	2,24	99,27	99,27
Mo IL-12(p70) (78)	UmAdMSCs 48h replicate 1	151,30	83,30	1,77	1,17	139,14	139,14
Mo IL-12(p70) (78)	UmAdMSCs 48h replicate 2	143,00	75,00	5,66	3,96	126,05	126,05
Mo IL-12(p70) (78)	UmAdMSCs 48h replicate 3	145,30	77,30	0,35	0,24	129,61	129,61
Mo IL-12(p70) (78)	UmAdMSCs 72h replicate 1	168,50	100,50	12,02	7,13	166,68	166,68
Mo IL-12(p70) (78)	UmAdMSCs 72h replicate 2	184,50	116,50	1,41	0,77	192,44	192,44
Mo IL-12(p70) (78)	UmAdMSCs 72h replicate 3	157,50	89,50	2,12	1,35	149,09	149,09
Mo IL-12(p70) (78)	FucmAdMSCs 0h replicate 1	75,50	7,50	2,12	2,81	21,47	21,47
Mo IL-12(p70) (78)	FucmAdMSCs 0h replicate 2	76,50	8,50	3,54	4,62	22,97	22,97
Mo IL-12(p70) (78)	FucmAdMSCs 0h replicate 3	72,30	4,30	0,35	0,49	16,57	16,57
Mo IL-12(p70) (78)	FucmAdMSCs 4h replicate 1	118,30	50,30	5,30	4,48	87,15	87,15
Mo IL-12(p70) (78)	FucmAdMSCs 4h replicate 2	111,00	43,00	7,07	6,37	75,86	75,86
Mo IL-12(p70) (78)	FucmAdMSCs 4h replicate 3	127,30	59,30	6,01	4,72	101,23	101,23
Mo IL-12(p70) (78)	FucmAdMSCs 12h replicate 1	140,50	72,50	3,54	2,52	122,10	122,10
Mo IL-12(p70) (78)	FucmAdMSCs 12h replicate 2	140,50	72,50	5,66	4,03	122,10	122,10
Mo IL-12(p70) (78)	FucmAdMSCs 12h replicate 3	147,50	79,50	0,71	0,48	133,18	133,18
Mo IL-12(p70) (78)	FucmAdMSCs 24h replicate 1	107,50	39,50	3,54	3,29	70,43	70,43

Mo IL-12(p70) (78)	FucmAdMSCs 24h replicate 2	122,30	54,30	5,30	4,34	93,40	93,40
Mo IL-12(p70) (78)	FucmAdMSCs 24h replicate 3	133,00	65,00	5,66	4,25	110,26	110,26
Mo IL-12(p70) (78)	FucmAdMSCs 48h replicate 1	121,00	53,00	5,66	4,68	91,44	91,44
Mo IL-12(p70) (78)	FucmAdMSCs 48h replicate 2	159,50	91,50	2,12	1,33	152,28	152,28
Mo IL-12(p70) (78)	FucmAdMSCs 48h replicate 3	164,50	96,50	7,78	4,73	160,27	160,27
Mo IL-12(p70) (78)	FucmAdMSCs 72h replicate 1	147,00	79,00	7,07	4,81	132,39	132,39
Mo IL-12(p70) (78)	FucmAdMSCs 72h replicate 2	177,00	109,00	1,41	0,80	180,34	180,34
Mo IL-12(p70) (78)	FucmAdMSCs 72h replicate 3	178,50	110,50	0,71	0,40	182,76	182,76
Mo IL-12(p70) (78)	R150	174,00	106,00	11,36	6,53	702,06	702,06
Mo IL-12(p70) (78)	R151	179,30	111,30	6,33	3,53	736,41	736,41
Mo IL-13 (37)	Reagent Name: Background0	8,50	8,50	0,71	8,32		
Mo IL-13 (37)	UmAdMSCs 0h replicate 1	9,50	1,00	0,71	7,44	OOR <	*6.59
Mo IL-13 (37)	UmAdMSCs 0h replicate 2	9,50	1,00	0,71	7,44	OOR <	*6.59
Mo IL-13 (37)	UmAdMSCs 0h replicate 3	9,00	0,50	0,00	0,00	OOR <	*2.87
Mo IL-13 (37)	UmAdMSCs 4h replicate 1	12,00	3,50	0,00	0,00	21,00	21,00
Mo IL-13 (37)	UmAdMSCs 4h replicate 2	15,00	6,50	0,00	0,00	35,43	35,43
Mo IL-13 (37)	UmAdMSCs 4h replicate 3	16,00	7,50	0,00	0,00	39,91	39,91
Mo IL-13 (37)	UmAdMSCs 12h replicate 1	16,50	8,00	0,71	4,29	42,11	42,11
Mo IL-13 (37)	UmAdMSCs 12h replicate 2	56,50	48,00	47,38	83,85	181,63	181,63
Mo IL-13 (37)	UmAdMSCs 12h replicate 3	25,50	17,00	0,71	2,77	78,19	78,19
Mo IL-13 (37)	UmAdMSCs 24h replicate 1	19,00	10,50	1,41	7,44	52,70	52,70
Mo IL-13 (37)	UmAdMSCs 24h replicate 2	17,50	9,00	0,71	4,04	46,41	46,41
Mo IL-13 (37)	UmAdMSCs 24h replicate 3	21,00	12,50	0,00	0,00	60,81	60,81
Mo IL-13 (37)	UmAdMSCs 48h replicate 1	28,00	19,50	0,00	0,00	87,44	87,44
Mo IL-13 (37)	UmAdMSCs 48h replicate 2	23,00	14,50	1,41	6,15	68,67	68,67
Mo IL-13 (37)	UmAdMSCs 48h replicate 3	26,00	17,50	1,41	5,44	80,06	80,06
Mo IL-13 (37)	UmAdMSCs 72h replicate 1	35,50	27,00	2,12	5,98	113,94	113,94
Mo IL-13 (37)	UmAdMSCs 72h replicate 2	39,50	31,00	0,71	1,79	127,45	127,45
Mo IL-13 (37)	UmAdMSCs 72h replicate 3	31,00	22,50	0,00	0,00	98,25	98,25
Mo IL-13 (37)	FucmAdMSCs 0h replicate 1	9,50	1,00	0,71	7,44	OOR <	*6.59
Mo IL-13 (37)	FucmAdMSCs 0h replicate 2	10,00	1,50	0,00	0,00	OOR <	*9.83
Mo IL-13 (37)	FucmAdMSCs 0h replicate 3	9,00	0,50	0,00	0,00	OOR <	*2.87
Mo IL-13 (37)	FucmAdMSCs 4h replicate 1	15,50	7,00	0,71	4,56	37,69	37,69
Mo IL-13 (37)	FucmAdMSCs 4h replicate 2	15,00	6,50	0,00	0,00	35,43	35,43
Mo IL-13 (37)	FucmAdMSCs 4h replicate 3	18,00	9,50	0,00	0,00	48,53	48,53
Mo IL-13 (37)	FucmAdMSCs 12h replicate 1	23,50	15,00	0,71	3,01	70,60	70,60
Mo IL-13 (37)	FucmAdMSCs 12h replicate 2	25,50	17,00	0,71	2,77	78,19	78,19
Mo IL-13 (37)	FucmAdMSCs 12h replicate 3	27,00	18,50	0,00	0,00	83,77	83,77
Mo IL-13 (37)	FucmAdMSCs 24h replicate 1	14,80	6,30	1,06	7,19	34,29	34,29
Mo IL-13 (37)	FucmAdMSCs 24h replicate 2	19,80	11,30	0,35	1,79	55,77	55,77
Mo IL-13 (37)	FucmAdMSCs 24h replicate 3	22,50	14,00	0,71	3,14	66,72	66,72
Mo IL-13 (37)	FucmAdMSCs 48h replicate 1	18,50	10,00	2,12	11,47	50,62	50,62
Mo IL-13 (37)	FucmAdMSCs 48h replicate 2	28,50	20,00	0,71	2,48	89,27	89,27
Mo IL-13 (37)	FucmAdMSCs 48h replicate 3	31,00	22,50	1,41	4,56	98,25	98,25
Mo IL-13 (37)	FucmAdMSCs 72h replicate 1	26,80	18,30	1,06	3,97	82,85	82,85
Mo IL-13 (37)	FucmAdMSCs 72h replicate 2	38,00	29,50	0,00	0,00	122,43	122,43
Mo IL-13 (37)	FucmAdMSCs 72h replicate 3	36,80	28,30	1,06	2,89	118,20	118,20
Mo IL-13 (37)	R150	39,30	30,80	4,73	12,01	507,59	507,59
Mo IL-13 (37)	R151	42,30	33,80	3,06	7,22	547,29	547,29
Mo IL-17 (72)	Reagent Name: Background0	20,00	20,00	0,00	0,00		
Mo IL-17 (72)	UmAdMSCs 0h replicate 1	28,00	8,00	1,41	5,05	2,95	2,95
Mo IL-17 (72)	UmAdMSCs 0h replicate 2	28,00	8,00	4,24	15,15	2,95	2,95
Mo IL-17 (72)	UmAdMSCs 0h replicate 3	27,30	7,30	0,35	1,30	2,70	2,70
Mo IL-17 (72)	UmAdMSCs 4h replicate 1	27,50	7,50	2,12	7,71	2,79	2,79
Mo IL-17 (72)	UmAdMSCs 4h replicate 2	30,30	10,30	1,06	3,51	3,66	3,66
Mo IL-17 (72)	UmAdMSCs 4h replicate 3	31,00	11,00	2,83	9,12	3,89	3,89
Mo IL-17 (72)	UmAdMSCs 12h replicate 1	29,50	9,50	0,71	2,40	3,42	3,42
Mo IL-17 (72)	UmAdMSCs 12h replicate 2	62,00	42,00	38,18	61,59	12,16	12,16
Mo IL-17 (72)	UmAdMSCs 12h replicate 3	35,50	15,50	0,71	1,99	5,22	5,22
Mo IL-17 (72)	UmAdMSCs 24h replicate 1	32,50	12,50	0,71	2,18	4,34	4,34
Mo IL-17 (72)	UmAdMSCs 24h replicate 2	31,00	11,00	1,41	4,56	3,89	3,89
Mo IL-17 (72)	UmAdMSCs 24h replicate 3	33,00	13,00	1,41	4,29	4,49	4,49
Mo IL-17 (72)	UmAdMSCs 48h replicate 1	37,00	17,00	0,00	0,00	5,65	5,65
Mo IL-17 (72)	UmAdMSCs 48h replicate 2	36,50	16,50	2,12	5,81	5,51	5,51
Mo IL-17 (72)	UmAdMSCs 48h replicate 3	34,30	14,30	0,35	1,03	4,86	4,86
Mo IL-17 (72)	UmAdMSCs 72h replicate 1	45,00	25,00	0,00	0,00	7,84	7,84
Mo IL-17 (72)	UmAdMSCs 72h replicate 2	46,00	26,00	0,00	0,00	8,11	8,11
Mo IL-17 (72)	UmAdMSCs 72h replicate 3	43,50	23,50	4,95	11,38	7,44	7,44
Mo IL-17 (72)	FucmAdMSCs 0h replicate 1	26,50	6,50	0,71	2,67	2,46	2,46
Mo IL-17 (72)	FucmAdMSCs 0h replicate 2	26,50	6,50	0,71	2,67	2,46	2,46

Mo IL-17 (72)	FucmAdMSCs 0h replicate 3	26,00	6,00	0,00	0,00	2,29	2,29
Mo IL-17 (72)	FucmAdMSCs 4h replicate 1	32,00	12,00	1,41	4,42	4,19	4,19
Mo IL-17 (72)	FucmAdMSCs 4h replicate 2	29,50	9,50	0,71	2,40	3,42	3,42
Mo IL-17 (72)	FucmAdMSCs 4h replicate 3	31,50	11,50	0,71	2,24	4,04	4,04
Mo IL-17 (72)	FucmAdMSCs 12h replicate 1	34,80	14,80	0,35	1,02	5,00	5,00
Mo IL-17 (72)	FucmAdMSCs 12h replicate 2	36,50	16,50	0,71	1,94	5,51	5,51
Mo IL-17 (72)	FucmAdMSCs 12h replicate 3	36,50	16,50	0,71	1,94	5,51	5,51
Mo IL-17 (72)	FucmAdMSCs 24h replicate 1	26,00	6,00	1,41	5,44	2,29	2,29
Mo IL-17 (72)	FucmAdMSCs 24h replicate 2	32,30	12,30	0,35	1,10	4,27	4,27
Mo IL-17 (72)	FucmAdMSCs 24h replicate 3	33,00	13,00	1,41	4,29	4,49	4,49
Mo IL-17 (72)	FucmAdMSCs 48h replicate 1	30,50	10,50	3,54	11,59	3,73	3,73
Mo IL-17 (72)	FucmAdMSCs 48h replicate 2	40,80	20,80	1,77	4,34	6,69	6,69
Mo IL-17 (72)	FucmAdMSCs 48h replicate 3	41,00	21,00	2,83	6,90	6,76	6,76
Mo IL-17 (72)	FucmAdMSCs 72h replicate 1	37,30	17,30	0,35	0,95	5,72	5,72
Mo IL-17 (72)	FucmAdMSCs 72h replicate 2	43,00	23,00	1,41	3,29	7,31	7,31
Mo IL-17 (72)	FucmAdMSCs 72h replicate 3	42,00	22,00	0,00	0,00	7,04	7,04
Mo IL-17 (72)	R150	405,80	385,80	32,20	7,93	314,53	314,53
Mo IL-17 (72)	R151	532,50	512,50	26,13	4,91	399,92	399,92
Mo Eotaxin (74)	Reagent Name: Background0	20,50	20,50	0,71	3,45		
Mo Eotaxin (74)	UmAdMSCs 0h replicate 1	35,50	15,00	14,85	41,83	36,89	36,89
Mo Eotaxin (74)	UmAdMSCs 0h replicate 2	24,50	4,00	2,12	8,66	14,65	14,65
Mo Eotaxin (74)	UmAdMSCs 0h replicate 3	24,50	4,00	0,71	2,89	14,65	14,65
Mo Eotaxin (74)	UmAdMSCs 4h replicate 1	57,00	36,50	1,41	2,48	72,26	72,26
Mo Eotaxin (74)	UmAdMSCs 4h replicate 2	106,50	86,00	0,71	0,66	140,24	140,24
Mo Eotaxin (74)	UmAdMSCs 4h replicate 3	145,80	125,30	3,18	2,18	188,02	188,02
Mo Eotaxin (74)	UmAdMSCs 12h replicate 1	755,50	735,00	20,51	2,71	753,20	753,20
Mo Eotaxin (74)	UmAdMSCs 12h replicate 2	1544,00	1523,50	100,41	6,50	1337,10	1337,10
Mo Eotaxin (74)	UmAdMSCs 12h replicate 3	1414,00	1393,50	74,95	5,30	1246,27	1246,27
Mo Eotaxin (74)	UmAdMSCs 24h replicate 1	1521,50	1501,00	98,29	6,46	1321,49	1321,49
Mo Eotaxin (74)	UmAdMSCs 24h replicate 2	1275,00	1254,50	128,69	10,09	1147,21	1147,21
Mo Eotaxin (74)	UmAdMSCs 24h replicate 3	1906,80	1886,30	96,52	5,06	1582,75	1582,75
Mo Eotaxin (74)	UmAdMSCs 48h replicate 1	8316,80	8296,30	122,68	1,48	5355,37	5355,37
Mo Eotaxin (74)	UmAdMSCs 48h replicate 2	5883,50	5863,00	342,95	5,83	3950,23	3950,23
Mo Eotaxin (74)	UmAdMSCs 48h replicate 3	8082,00	8061,50	11,31	0,14	5216,21	5216,21
Mo Eotaxin (74)	UmAdMSCs 72h replicate 1	13554,00	13533,50	8,49	0,06	9127,81	9127,81
Mo Eotaxin (74)	UmAdMSCs 72h replicate 2	14259,00	14238,50	7,07	0,05	9839,83	9839,83
Mo Eotaxin (74)	UmAdMSCs 72h replicate 3	12654,50	12634,00	348,60	2,75	8328,43	8328,43
Mo Eotaxin (74)	FucmAdMSCs 0h replicate 1	24,50	4,00	0,71	2,89	14,65	14,65
Mo Eotaxin (74)	FucmAdMSCs 0h replicate 2	31,00	10,50	11,31	36,50	28,41	28,41
Mo Eotaxin (74)	FucmAdMSCs 0h replicate 3	22,50	2,00	0,71	3,14	9,72	9,72
Mo Eotaxin (74)	FucmAdMSCs 4h replicate 1	135,00	114,50	0,00	0,00	175,30	175,30
Mo Eotaxin (74)	FucmAdMSCs 4h replicate 2	169,00	148,50	0,00	0,00	214,78	214,78
Mo Eotaxin (74)	FucmAdMSCs 4h replicate 3	226,00	205,50	11,31	5,01	276,97	276,97
Mo Eotaxin (74)	FucmAdMSCs 12h replicate 1	1897,50	1877,00	3,54	0,19	1576,61	1576,61
Mo Eotaxin (74)	FucmAdMSCs 12h replicate 2	2314,80	2294,30	55,51	2,40	1848,17	1848,17
Mo Eotaxin (74)	FucmAdMSCs 12h replicate 3	2390,50	2370,00	122,33	5,12	1896,41	1896,41
Mo Eotaxin (74)	FucmAdMSCs 24h replicate 1	1019,50	999,00	82,73	8,11	958,87	958,87
Mo Eotaxin (74)	FucmAdMSCs 24h replicate 2	2111,30	2090,80	149,55	7,08	1717,04	1717,04
Mo Eotaxin (74)	FucmAdMSCs 24h replicate 3	4669,30	4648,80	182,08	3,90	3257,93	3257,93
Mo Eotaxin (74)	FucmAdMSCs 48h replicate 1	6677,50	6657,00	111,02	1,66	4402,36	4402,36
Mo Eotaxin (74)	FucmAdMSCs 48h replicate 2	9956,80	9936,30	25,81	0,26	6369,61	6369,61
Mo Eotaxin (74)	FucmAdMSCs 48h replicate 3	12068,00	12047,50	553,66	4,59	7856,50	7856,50
Mo Eotaxin (74)	FucmAdMSCs 72h replicate 1	11940,80	11920,30	302,29	2,53	7758,29	7758,29
Mo Eotaxin (74)	FucmAdMSCs 72h replicate 2	14916,30	14895,80	133,29	0,89	10597,39	10597,39
Mo Eotaxin (74)	FucmAdMSCs 72h replicate 3	15535,80	15515,30	191,27	1,23	11425,89	11425,89
Mo Eotaxin (74)	R150	839,80	819,30	73,01	8,69	3281,47	3281,47
Mo Eotaxin (74)	R151	1078,30	1057,80	57,52	5,33	4012,19	4012,19
Mo G-CSF (54)	Reagent Name: Background0	26,00	26,00	0,00	0,00		
Mo G-CSF (54)	UmAdMSCs 0h replicate 1	31,00	5,00	1,41	4,56	4,06	4,06
Mo G-CSF (54)	UmAdMSCs 0h replicate 2	29,80	3,80	1,77	5,94	3,55	3,55
Mo G-CSF (54)	UmAdMSCs 0h replicate 3	29,00	3,00	0,00	0,00	3,24	3,24
Mo G-CSF (54)	UmAdMSCs 4h replicate 1	34,50	8,50	3,54	10,25	5,46	5,46
Mo G-CSF (54)	UmAdMSCs 4h replicate 2	38,50	12,50	0,71	1,84	7,02	7,02
Mo G-CSF (54)	UmAdMSCs 4h replicate 3	40,30	14,30	1,06	2,64	7,70	7,70
Mo G-CSF (54)	UmAdMSCs 12h replicate 1	39,50	13,50	2,12	5,37	7,41	7,41
Mo G-CSF (54)	UmAdMSCs 12h replicate 2	90,50	64,50	48,79	53,91	25,78	25,78
Mo G-CSF (54)	UmAdMSCs 12h replicate 3	59,00	33,00	0,00	0,00	14,66	14,66
Mo G-CSF (54)	UmAdMSCs 24h replicate 1	50,80	24,80	3,89	7,66	11,64	11,64
Mo G-CSF (54)	UmAdMSCs 24h replicate 2	44,50	18,50	0,71	1,59	9,31	9,31
Mo G-CSF (54)	UmAdMSCs 24h replicate 3	49,80	23,80	1,06	2,13	11,27	11,27

Mo G-CSF (54)	UmAdMSCs 48h replicate 1	63,00	37,00	2,83	4,49	16,10	16,10
Mo G-CSF (54)	UmAdMSCs 48h replicate 2	58,00	32,00	1,41	2,44	14,30	14,30
Mo G-CSF (54)	UmAdMSCs 48h replicate 3	67,00	41,00	1,41	2,11	17,53	17,53
Mo G-CSF (54)	UmAdMSCs 72h replicate 1	86,50	60,50	3,54	4,09	24,39	24,39
Mo G-CSF (54)	UmAdMSCs 72h replicate 2	241,80	215,80	0,35	0,15	75,68	75,68
Mo G-CSF (54)	UmAdMSCs 72h replicate 3	92,00	66,00	4,24	4,61	26,30	26,30
Mo G-CSF (54)	FucmAdMSCs 0h replicate 1	28,00	2,00	0,00	0,00	2,82	2,82
Mo G-CSF (54)	FucmAdMSCs 0h replicate 2	28,00	2,00	0,00	0,00	2,82	2,82
Mo G-CSF (54)	FucmAdMSCs 0h replicate 3	27,00	1,00	1,41	5,24	2,40	2,40
Mo G-CSF (54)	FucmAdMSCs 4h replicate 1	43,50	17,50	1,41	3,25	8,93	8,93
Mo G-CSF (54)	FucmAdMSCs 4h replicate 2	40,00	14,00	0,00	0,00	7,60	7,60
Mo G-CSF (54)	FucmAdMSCs 4h replicate 3	49,00	23,00	0,00	0,00	10,99	10,99
Mo G-CSF (54)	FucmAdMSCs 12h replicate 1	60,80	34,80	1,77	2,91	15,29	15,29
Mo G-CSF (54)	FucmAdMSCs 12h replicate 2	67,00	41,00	0,71	1,06	17,53	17,53
Mo G-CSF (54)	FucmAdMSCs 12h replicate 3	66,00	40,00	4,24	6,43	17,18	17,18
Mo G-CSF (54)	FucmAdMSCs 24h replicate 1	38,50	12,50	0,71	1,84	7,02	7,02
Mo G-CSF (54)	FucmAdMSCs 24h replicate 2	50,00	24,00	2,83	5,66	11,36	11,36
Mo G-CSF (54)	FucmAdMSCs 24h replicate 3	56,50	30,50	2,12	3,75	13,75	13,75
Mo G-CSF (54)	FucmAdMSCs 48h replicate 1	48,00	22,00	2,83	5,89	10,62	10,62
Mo G-CSF (54)	FucmAdMSCs 48h replicate 2	73,80	47,80	0,35	0,48	19,93	19,93
Mo G-CSF (54)	FucmAdMSCs 48h replicate 3	77,50	51,50	6,36	8,21	21,25	21,25
Mo G-CSF (54)	FucmAdMSCs 72h replicate 1	68,80	42,80	0,35	0,51	18,16	18,16
Mo G-CSF (54)	FucmAdMSCs 72h replicate 2	100,30	74,30	1,06	1,06	29,13	29,13
Mo G-CSF (54)	FucmAdMSCs 72h replicate 3	97,00	71,00	5,66	5,83	28,02	28,02
Mo G-CSF (54)	R150	93,30	67,30	3,79	4,06	107,02	107,02
Mo G-CSF (54)	R151	112,20	86,20	2,25	2,01	132,77	132,77
Mo GM-CSF (73)	Reagent Name: Background0	16,80	16,80	0,35	2,11		
Mo GM-CSF (73)	UmAdMSCs 0h replicate 1	20,00	3,30	0,00	0,00	OOR <	*23.61
Mo GM-CSF (73)	UmAdMSCs 0h replicate 2	19,00	2,30	1,41	7,44	OOR <	*16.49
Mo GM-CSF (73)	UmAdMSCs 0h replicate 3	19,00	2,30	1,41	7,44	OOR <	*16.49
Mo GM-CSF (73)	UmAdMSCs 4h replicate 1	22,00	5,30	1,41	6,43	33,64	33,64
Mo GM-CSF (73)	UmAdMSCs 4h replicate 2	26,00	9,30	0,00	0,00	47,82	47,82
Mo GM-CSF (73)	UmAdMSCs 4h replicate 3	26,50	9,80	0,71	2,67	49,31	49,31
Mo GM-CSF (73)	UmAdMSCs 12h replicate 1	25,50	8,80	2,12	8,32	46,28	46,28
Mo GM-CSF (73)	UmAdMSCs 12h replicate 2	70,00	53,30	53,74	76,77	123,77	123,77
Mo GM-CSF (73)	UmAdMSCs 12h replicate 3	35,50	18,80	0,71	1,99	71,10	71,10
Mo GM-CSF (73)	UmAdMSCs 24h replicate 1	30,00	13,30	1,41	4,71	58,72	58,72
Mo GM-CSF (73)	UmAdMSCs 24h replicate 2	27,50	10,80	0,71	2,57	52,17	52,17
Mo GM-CSF (73)	UmAdMSCs 24h replicate 3	31,00	14,30	0,00	0,00	61,15	61,15
Mo GM-CSF (73)	UmAdMSCs 48h replicate 1	37,00	20,30	1,41	3,82	74,13	74,13
Mo GM-CSF (73)	UmAdMSCs 48h replicate 2	33,50	16,80	3,54	10,55	66,86	66,86
Mo GM-CSF (73)	UmAdMSCs 48h replicate 3	35,00	18,30	1,41	4,04	70,06	70,06
Mo GM-CSF (73)	UmAdMSCs 72h replicate 1	47,00	30,30	4,24	9,03	91,90	91,90
Mo GM-CSF (73)	UmAdMSCs 72h replicate 2	51,50	34,80	2,12	4,12	98,90	98,90
Mo GM-CSF (73)	UmAdMSCs 72h replicate 3	42,00	25,30	0,00	0,00	83,47	83,47
Mo GM-CSF (73)	FucmAdMSCs 0h replicate 1	18,50	1,80	0,71	3,82	OOR <	*11.39
Mo GM-CSF (73)	FucmAdMSCs 0h replicate 2	19,00	2,30	0,00	0,00	OOR <	*16.49
Mo GM-CSF (73)	FucmAdMSCs 0h replicate 3	18,50	1,80	0,71	3,82	OOR <	*11.39
Mo GM-CSF (73)	FucmAdMSCs 4h replicate 1	26,50	9,80	0,71	2,67	49,31	49,31
Mo GM-CSF (73)	FucmAdMSCs 4h replicate 2	24,30	7,50	1,06	4,37	42,19	42,19
Mo GM-CSF (73)	FucmAdMSCs 4h replicate 3	29,50	12,80	2,12	7,19	57,47	57,47
Mo GM-CSF (73)	FucmAdMSCs 12h replicate 1	33,50	16,80	0,71	2,11	66,86	66,86
Mo GM-CSF (73)	FucmAdMSCs 12h replicate 2	36,50	19,80	0,71	1,94	73,14	73,14
Mo GM-CSF (73)	FucmAdMSCs 12h replicate 3	35,50	18,80	0,71	1,99	71,10	71,10
Mo GM-CSF (73)	FucmAdMSCs 24h replicate 1	23,00	6,30	0,00	0,00	37,67	37,67
Mo GM-CSF (73)	FucmAdMSCs 24h replicate 2	28,50	11,80	2,12	7,44	54,88	54,88
Mo GM-CSF (73)	FucmAdMSCs 24h replicate 3	30,50	13,80	0,71	2,32	59,95	59,95
Mo GM-CSF (73)	FucmAdMSCs 48h replicate 1	27,80	11,00	1,77	6,37	52,86	52,86
Mo GM-CSF (73)	FucmAdMSCs 48h replicate 2	41,00	24,30	2,83	6,90	81,68	81,68
Mo GM-CSF (73)	FucmAdMSCs 48h replicate 3	42,00	25,30	2,83	6,73	83,47	83,47
Mo GM-CSF (73)	FucmAdMSCs 72h replicate 1	35,00	18,30	1,41	4,04	70,06	70,06
Mo GM-CSF (73)	FucmAdMSCs 72h replicate 2	47,00	30,30	0,00	0,00	91,90	91,90
Mo GM-CSF (73)	FucmAdMSCs 72h replicate 3	44,50	27,80	0,71	1,59	87,78	87,78
Mo GM-CSF (73)	R150	68,80	52,10	5,48	7,97	489,38	489,38
Mo GM-CSF (73)	R151	78,30	61,60	7,78	9,94	534,23	534,23
Mo IFN-g (34)	Reagent Name: Background0	19,50	19,50	0,71	3,63		
Mo IFN-g (34)	UmAdMSCs 0h replicate 1	24,00	4,50	0,00	0,00	3,74	3,74
Mo IFN-g (34)	UmAdMSCs 0h replicate 2	24,00	4,50	1,41	5,89	3,74	3,74
Mo IFN-g (34)	UmAdMSCs 0h replicate 3	23,50	4,00	2,12	9,03	3,50	3,50
Mo IFN-g (34)	UmAdMSCs 4h replicate 1	26,00	6,50	1,41	5,44	4,69	4,69

Mo IFN-g (34)	UmAdMSCs 4h replicate 2	27,00	7,50	0,00	0,00	5,17	5,17
Mo IFN-g (34)	UmAdMSCs 4h replicate 3	30,50	11,00	0,71	2,32	6,81	6,81
Mo IFN-g (34)	UmAdMSCs 12h replicate 1	29,00	9,50	1,41	4,88	6,11	6,11
Mo IFN-g (34)	UmAdMSCs 12h replicate 2	61,50	42,00	36,06	58,64	20,77	20,77
Mo IFN-g (34)	UmAdMSCs 12h replicate 3	39,00	19,50	1,41	3,63	10,71	10,71
Mo IFN-g (34)	UmAdMSCs 24h replicate 1	34,00	14,50	1,41	4,16	8,43	8,43
Mo IFN-g (34)	UmAdMSCs 24h replicate 2	33,00	13,50	0,00	0,00	7,96	7,96
Mo IFN-g (34)	UmAdMSCs 24h replicate 3	38,00	18,50	0,00	0,00	10,26	10,26
Mo IFN-g (34)	UmAdMSCs 48h replicate 1	40,80	21,30	2,47	6,07	11,51	11,51
Mo IFN-g (34)	UmAdMSCs 48h replicate 2	37,00	17,50	1,41	3,82	9,80	9,80
Mo IFN-g (34)	UmAdMSCs 48h replicate 3	37,50	18,00	0,71	1,89	10,03	10,03
Mo IFN-g (34)	UmAdMSCs 72h replicate 1	44,00	24,50	2,83	6,43	12,98	12,98
Mo IFN-g (34)	UmAdMSCs 72h replicate 2	46,00	26,50	0,00	0,00	13,88	13,88
Mo IFN-g (34)	UmAdMSCs 72h replicate 3	41,50	22,00	3,54	8,52	11,85	11,85
Mo IFN-g (34)	FucmAdMSCs 0h replicate 1	23,80	4,30	0,35	1,49	3,62	3,62
Mo IFN-g (34)	FucmAdMSCs 0h replicate 2	23,50	4,00	0,71	3,01	3,50	3,50
Mo IFN-g (34)	FucmAdMSCs 0h replicate 3	23,00	3,50	1,41	6,15	3,26	3,26
Mo IFN-g (34)	FucmAdMSCs 4h replicate 1	31,00	11,50	0,00	0,00	7,04	7,04
Mo IFN-g (34)	FucmAdMSCs 4h replicate 2	26,00	6,50	1,41	5,44	4,69	4,69
Mo IFN-g (34)	FucmAdMSCs 4h replicate 3	33,00	13,50	1,41	4,29	7,96	7,96
Mo IFN-g (34)	FucmAdMSCs 12h replicate 1	34,50	15,00	1,41	4,10	8,66	8,66
Mo IFN-g (34)	FucmAdMSCs 12h replicate 2	36,00	16,50	0,00	0,00	9,34	9,34
Mo IFN-g (34)	FucmAdMSCs 12h replicate 3	37,50	18,00	0,71	1,89	10,03	10,03
Mo IFN-g (34)	FucmAdMSCs 24h replicate 1	30,50	11,00	3,54	11,59	6,81	6,81
Mo IFN-g (34)	FucmAdMSCs 24h replicate 2	35,00	15,50	0,00	0,00	8,89	8,89
Mo IFN-g (34)	FucmAdMSCs 24h replicate 3	35,50	16,00	0,71	1,99	9,12	9,12
Mo IFN-g (34)	FucmAdMSCs 48h replicate 1	32,00	12,50	1,41	4,42	7,50	7,50
Mo IFN-g (34)	FucmAdMSCs 48h replicate 2	41,50	22,00	0,71	1,70	11,85	11,85
Mo IFN-g (34)	FucmAdMSCs 48h replicate 3	42,50	23,00	3,54	8,32	12,30	12,30
Mo IFN-g (34)	FucmAdMSCs 72h replicate 1	40,00	20,50	0,00	0,00	11,17	11,17
Mo IFN-g (34)	FucmAdMSCs 72h replicate 2	43,50	24,00	0,71	1,63	12,75	12,75
Mo IFN-g (34)	FucmAdMSCs 72h replicate 3	42,00	22,50	1,41	3,37	12,08	12,08
Mo IFN-g (34)	R150	94,50	75,00	8,53	9,03	140,50	140,50
Mo IFN-g (34)	R151	108,30	88,80	4,93	4,55	164,21	164,21
Mo KC (57)	Reagent Name: Background0	12,00	12,00	0,00	0,00		
Mo KC (57)	UmAdMSCs 0h replicate 1	15,50	3,50	4,95	31,93	2,71	2,71
Mo KC (57)	UmAdMSCs 0h replicate 2	12,00	0,00	1,41	11,79	OOR <	*0.74
Mo KC (57)	UmAdMSCs 0h replicate 3	12,50	0,50	0,71	5,66	OOR <	*1.07
Mo KC (57)	UmAdMSCs 4h replicate 1	181,00	169,00	2,83	1,56	48,03	48,03
Mo KC (57)	UmAdMSCs 4h replicate 2	387,30	375,30	9,55	2,47	89,83	89,83
Mo KC (57)	UmAdMSCs 4h replicate 3	570,30	558,30	13,08	2,29	122,94	122,94
Mo KC (57)	UmAdMSCs 12h replicate 1	495,00	483,00	4,24	0,86	109,63	109,63
Mo KC (57)	UmAdMSCs 12h replicate 2	924,00	912,00	41,01	4,44	181,73	181,73
Mo KC (57)	UmAdMSCs 12h replicate 3	953,80	941,80	41,37	4,34	186,46	186,46
Mo KC (57)	UmAdMSCs 24h replicate 1	686,00	674,00	38,18	5,57	142,77	142,77
Mo KC (57)	UmAdMSCs 24h replicate 2	541,80	529,80	55,51	10,25	117,94	117,94
Mo KC (57)	UmAdMSCs 24h replicate 3	804,50	792,50	38,89	4,83	162,43	162,43
Mo KC (57)	UmAdMSCs 48h replicate 1	1268,50	1256,50	55,15	4,35	235,21	235,21
Mo KC (57)	UmAdMSCs 48h replicate 2	1078,50	1066,50	75,66	7,02	206,05	206,05
Mo KC (57)	UmAdMSCs 48h replicate 3	1646,80	1634,80	18,03	1,09	291,46	291,46
Mo KC (57)	UmAdMSCs 72h replicate 1	2863,30	2851,30	110,66	3,86	464,29	464,29
Mo KC (57)	UmAdMSCs 72h replicate 2	5421,50	5409,50	284,96	5,26	827,49	827,49
Mo KC (57)	UmAdMSCs 72h replicate 3	2830,00	2818,00	8,49	0,30	459,64	459,64
Mo KC (57)	FucmAdMSCs 0h replicate 1	12,00	0,00	0,00	0,00	OOR <	*0.74
Mo KC (57)	FucmAdMSCs 0h replicate 2	15,00	3,00	4,24	28,28	2,46	2,46
Mo KC (57)	FucmAdMSCs 0h replicate 3	12,00	0,00	0,00	0,00	OOR <	*0.74
Mo KC (57)	FucmAdMSCs 4h replicate 1	450,00	438,00	8,49	1,89	101,48	101,48
Mo KC (57)	FucmAdMSCs 4h replicate 2	811,00	799,00	14,14	1,74	163,49	163,49
Mo KC (57)	FucmAdMSCs 4h replicate 3	943,50	931,50	19,09	2,02	184,83	184,83
Mo KC (57)	FucmAdMSCs 12h replicate 1	1214,80	1202,80	2,47	0,20	227,04	227,04
Mo KC (57)	FucmAdMSCs 12h replicate 2	1403,80	1391,80	20,15	1,44	255,56	255,56
Mo KC (57)	FucmAdMSCs 12h replicate 3	1348,30	1336,30	9,55	0,71	247,25	247,25
Mo KC (57)	FucmAdMSCs 24h replicate 1	378,80	366,80	13,08	3,45	88,22	88,22
Mo KC (57)	FucmAdMSCs 24h replicate 2	846,50	834,50	40,31	4,76	169,26	169,26
Mo KC (57)	FucmAdMSCs 24h replicate 3	1432,30	1420,30	63,29	4,42	259,81	259,81
Mo KC (57)	FucmAdMSCs 48h replicate 1	935,00	923,00	65,05	6,96	183,48	183,48
Mo KC (57)	FucmAdMSCs 48h replicate 2	1804,50	1792,50	13,44	0,74	314,40	314,40
Mo KC (57)	FucmAdMSCs 48h replicate 3	2232,30	2220,30	193,39	8,66	375,60	375,60
Mo KC (57)	FucmAdMSCs 72h replicate 1	1664,50	1652,50	68,59	4,12	294,05	294,05
Mo KC (57)	FucmAdMSCs 72h replicate 2	3714,50	3702,50	44,55	1,20	583,23	583,23

Mo KC (57)	FucmAdMSCs 72h replicate 3	3834,00	3822,00	135,76	3,54	599,99	599,99
Mo KC (57)	R150	124,30	112,30	3,21	2,59	139,68	139,68
Mo KC (57)	R151	140,50	128,50	11,26	8,01	155,12	155,12
Mo MCP-1 (51)	Reagent Name: Background0	9,00	9,00	1,41	15,71		
Mo MCP-1 (51)	UmAdMSCs 0h replicate 1	18,50	9,50	12,02	64,98	196,69	196,69
Mo MCP-1 (51)	UmAdMSCs 0h replicate 2	9,50	0,50	0,71	7,44	32,99	32,99
Mo MCP-1 (51)	UmAdMSCs 0h replicate 3	10,00	1,00	0,00	0,00	46,11	46,11
Mo MCP-1 (51)	UmAdMSCs 4h replicate 1	275,50	266,50	3,54	1,28	2207,46	2207,46
Mo MCP-1 (51)	UmAdMSCs 4h replicate 2	534,50	525,50	31,82	5,95	3629,78	3629,78
Mo MCP-1 (51)	UmAdMSCs 4h replicate 3	772,30	763,30	47,02	6,09	4771,55	4771,55
Mo MCP-1 (51)	UmAdMSCs 12h replicate 1	2054,80	2045,80	144,60	7,04	9829,03	9829,03
Mo MCP-1 (51)	UmAdMSCs 12h replicate 2	2587,30	2578,30	9,55	0,37	11645,70	11645,70
Mo MCP-1 (51)	UmAdMSCs 12h replicate 3	2596,00	2587,00	57,98	2,23	11674,66	11674,66
Mo MCP-1 (51)	UmAdMSCs 24h replicate 1	1767,30	1758,30	120,56	6,82	8796,22	8796,22
Mo MCP-1 (51)	UmAdMSCs 24h replicate 2	1651,50	1642,50	252,44	15,29	8367,89	8367,89
Mo MCP-1 (51)	UmAdMSCs 24h replicate 3	1982,30	1973,30	52,68	2,66	9572,45	9572,45
Mo MCP-1 (51)	UmAdMSCs 48h replicate 1	4037,00	4028,00	8,49	0,21	16151,68	16151,68
Mo MCP-1 (51)	UmAdMSCs 48h replicate 2	3092,80	3083,80	96,52	3,12	13279,07	13279,07
Mo MCP-1 (51)	UmAdMSCs 48h replicate 3	4579,50	4570,50	34,65	0,76	17719,36	17719,36
Mo MCP-1 (51)	UmAdMSCs 72h replicate 1	6617,00	6608,00	87,68	1,33	23218,30	23218,30
Mo MCP-1 (51)	UmAdMSCs 72h replicate 2	9486,00	9477,00	35,36	0,37	OOR >	*30244.40
Mo MCP-1 (51)	UmAdMSCs 72h replicate 3	6712,30	6703,30	140,36	2,09	23463,20	23463,20
Mo MCP-1 (51)	FucmAdMSCs 0h replicate 1	9,80	0,80	0,35	3,63	39,75	39,75
Mo MCP-1 (51)	FucmAdMSCs 0h replicate 2	14,30	5,30	7,42	52,10	130,11	130,11
Mo MCP-1 (51)	FucmAdMSCs 0h replicate 3	10,00	1,00	0,00	0,00	46,11	46,11
Mo MCP-1 (51)	FucmAdMSCs 4h replicate 1	1163,00	1154,00	14,14	1,22	6460,21	6460,21
Mo MCP-1 (51)	FucmAdMSCs 4h replicate 2	1386,00	1377,00	19,80	1,43	7353,38	7353,38
Mo MCP-1 (51)	FucmAdMSCs 4h replicate 3	1941,50	1932,50	3,54	0,18	9427,13	9427,13
Mo MCP-1 (51)	FucmAdMSCs 12h replicate 1	3088,00	3079,00	103,24	3,34	13264,07	13264,07
Mo MCP-1 (51)	FucmAdMSCs 12h replicate 2	3596,80	3587,80	95,81	2,66	14837,65	14837,65
Mo MCP-1 (51)	FucmAdMSCs 12h replicate 3	4161,00	4152,00	0,00	0,00	16514,73	16514,73
Mo MCP-1 (51)	FucmAdMSCs 24h replicate 1	1300,00	1291,00	174,66	13,44	7013,87	7013,87
Mo MCP-1 (51)	FucmAdMSCs 24h replicate 2	2353,50	2344,50	16,26	0,69	10861,94	10861,94
Mo MCP-1 (51)	FucmAdMSCs 24h replicate 3	3784,50	3775,50	125,16	3,31	15403,01	15403,01
Mo MCP-1 (51)	FucmAdMSCs 48h replicate 1	2977,30	2968,30	274,71	9,23	12912,59	12912,59
Mo MCP-1 (51)	FucmAdMSCs 48h replicate 2	5052,00	5043,00	110,31	2,18	19044,60	19044,60
Mo MCP-1 (51)	FucmAdMSCs 48h replicate 3	5482,80	5473,80	117,73	2,15	20224,09	20224,09
Mo MCP-1 (51)	FucmAdMSCs 72h replicate 1	5323,80	5314,80	12,37	0,23	19791,70	19791,70
Mo MCP-1 (51)	FucmAdMSCs 72h replicate 2	7425,50	7416,50	26,16	0,35	25268,71	25268,71
Mo MCP-1 (51)	FucmAdMSCs 72h replicate 3	7364,30	7355,30	114,90	1,56	25115,54	25115,54
Mo MCP-1 (51)	R150	66,80	57,80	5,25	7,86	2890,83	2890,83
Mo MCP-1 (51)	R151	73,80	64,80	6,79	9,19	3141,88	3141,88
Mo MIP-1a (77)	Reagent Name: Background0	21,00	21,00	0,00	0,00		
Mo MIP-1a (77)	UmAdMSCs 0h replicate 1	29,50	8,50	2,12	7,19	1,08	1,08
Mo MIP-1a (77)	UmAdMSCs 0h replicate 2	27,50	6,50	2,12	7,71	0,87	0,87
Mo MIP-1a (77)	UmAdMSCs 0h replicate 3	27,50	6,50	0,71	2,57	0,87	0,87
Mo MIP-1a (77)	UmAdMSCs 4h replicate 1	81,00	60,00	0,00	0,00	5,81	5,81
Mo MIP-1a (77)	UmAdMSCs 4h replicate 2	100,00	79,00	0,00	0,00	7,41	7,41
Mo MIP-1a (77)	UmAdMSCs 4h replicate 3	112,50	91,50	2,12	1,89	8,44	8,44
Mo MIP-1a (77)	UmAdMSCs 12h replicate 1	240,50	219,50	19,09	7,94	18,38	18,38
Mo MIP-1a (77)	UmAdMSCs 12h replicate 2	329,00	308,00	35,36	10,75	24,89	24,89
Mo MIP-1a (77)	UmAdMSCs 12h replicate 3	320,00	299,00	9,90	3,09	24,23	24,23
Mo MIP-1a (77)	UmAdMSCs 24h replicate 1	246,80	225,80	16,62	6,73	18,85	18,85
Mo MIP-1a (77)	UmAdMSCs 24h replicate 2	215,80	194,80	8,13	3,77	16,52	16,52
Mo MIP-1a (77)	UmAdMSCs 24h replicate 3	258,00	237,00	19,80	7,67	19,69	19,69
Mo MIP-1a (77)	UmAdMSCs 48h replicate 1	473,50	452,50	21,92	4,63	35,14	35,14
Mo MIP-1a (77)	UmAdMSCs 48h replicate 2	385,80	364,80	8,13	2,11	28,96	28,96
Mo MIP-1a (77)	UmAdMSCs 48h replicate 3	495,50	474,50	2,12	0,43	36,68	36,68
Mo MIP-1a (77)	UmAdMSCs 72h replicate 1	707,50	686,50	20,51	2,90	51,18	51,18
Mo MIP-1a (77)	UmAdMSCs 72h replicate 2	1826,00	1805,00	94,75	5,19	124,52	124,52
Mo MIP-1a (77)	UmAdMSCs 72h replicate 3	720,30	699,30	26,52	3,68	52,04	52,04
Mo MIP-1a (77)	FucmAdMSCs 0h replicate 1	28,00	7,00	1,41	5,05	0,92	0,92
Mo MIP-1a (77)	FucmAdMSCs 0h replicate 2	29,50	8,50	2,12	7,19	1,08	1,08
Mo MIP-1a (77)	FucmAdMSCs 0h replicate 3	26,00	5,00	0,00	0,00	OOR <	*0.71
Mo MIP-1a (77)	FucmAdMSCs 4h replicate 1	154,00	133,00	2,83	1,84	11,76	11,76
Mo MIP-1a (77)	FucmAdMSCs 4h replicate 2	152,30	131,30	4,60	3,02	11,62	11,62
Mo MIP-1a (77)	FucmAdMSCs 4h replicate 3	186,50	165,50	10,61	5,69	14,29	14,29
Mo MIP-1a (77)	FucmAdMSCs 12h replicate 1	427,30	406,30	8,13	1,90	31,90	31,90
Mo MIP-1a (77)	FucmAdMSCs 12h replicate 2	467,50	446,50	0,71	0,15	34,72	34,72
Mo MIP-1a (77)	FucmAdMSCs 12h replicate 3	476,50	455,50	13,44	2,82	35,35	35,35

Mo MIP-1a (77)	FucmAdMSCs 24h replicate 1	223,80	202,80	9,55	4,27	17,13	17,13
Mo MIP-1a (77)	FucmAdMSCs 24h replicate 2	337,00	316,00	28,28	8,39	25,46	25,46
Mo MIP-1a (77)	FucmAdMSCs 24h replicate 3	381,50	360,50	19,09	5,00	28,65	28,65
Mo MIP-1a (77)	FucmAdMSCs 48h replicate 1	373,00	352,00	9,19	2,46	28,05	28,05
Mo MIP-1a (77)	FucmAdMSCs 48h replicate 2	627,80	606,80	1,77	0,28	45,77	45,77
Mo MIP-1a (77)	FucmAdMSCs 48h replicate 3	629,00	608,00	45,25	7,19	45,86	45,86
Mo MIP-1a (77)	FucmAdMSCs 72h replicate 1	579,30	558,30	17,32	2,99	42,46	42,46
Mo MIP-1a (77)	FucmAdMSCs 72h replicate 2	893,30	872,30	12,37	1,39	63,61	63,61
Mo MIP-1a (77)	FucmAdMSCs 72h replicate 3	851,80	830,80	58,34	6,85	60,85	60,85
Mo MIP-1a (77)	R150	73,20	52,20	7,82	10,68	20,54	20,54
Mo MIP-1a (77)	R151	76,20	55,20	6,93	9,10	21,58	21,58
Mo MIP-1b (75)	Reagent Name: Background0	14,00	14,00	0,00	0,00		
Mo MIP-1b (75)	UmAdMSCs 0h replicate 1	15,50	1,50	0,71	4,56	OOR <	*1.80
Mo MIP-1b (75)	UmAdMSCs 0h replicate 2	15,00	1,00	1,41	9,43	OOR <	*1.41
Mo MIP-1b (75)	UmAdMSCs 0h replicate 3	14,50	0,50	0,71	4,88	OOR <	*0.98
Mo MIP-1b (75)	UmAdMSCs 4h replicate 1	22,00	8,00	0,00	0,00	5,88	5,88
Mo MIP-1b (75)	UmAdMSCs 4h replicate 2	27,00	13,00	0,00	0,00	8,50	8,50
Mo MIP-1b (75)	UmAdMSCs 4h replicate 3	30,30	16,30	0,35	1,17	10,09	10,09
Mo MIP-1b (75)	UmAdMSCs 12h replicate 1	45,30	31,30	0,35	0,78	16,75	16,75
Mo MIP-1b (75)	UmAdMSCs 12h replicate 2	102,30	88,30	41,37	40,46	37,84	37,84
Mo MIP-1b (75)	UmAdMSCs 12h replicate 3	69,50	55,50	0,71	1,02	26,25	26,25
Mo MIP-1b (75)	UmAdMSCs 24h replicate 1	52,50	38,50	3,54	6,73	19,71	19,71
Mo MIP-1b (75)	UmAdMSCs 24h replicate 2	45,80	31,80	1,77	3,86	16,96	16,96
Mo MIP-1b (75)	UmAdMSCs 24h replicate 3	54,50	40,50	2,12	3,89	20,51	20,51
Mo MIP-1b (75)	UmAdMSCs 48h replicate 1	82,00	68,00	5,66	6,90	30,80	30,80
Mo MIP-1b (75)	UmAdMSCs 48h replicate 2	67,50	53,50	6,36	9,43	25,51	25,51
Mo MIP-1b (75)	UmAdMSCs 48h replicate 3	79,00	65,00	1,41	1,79	29,73	29,73
Mo MIP-1b (75)	UmAdMSCs 72h replicate 1	116,50	102,50	4,95	4,25	42,60	42,60
Mo MIP-1b (75)	UmAdMSCs 72h replicate 2	139,00	125,00	7,07	5,09	49,88	49,88
Mo MIP-1b (75)	UmAdMSCs 72h replicate 3	101,50	87,50	2,12	2,09	37,59	37,59
Mo MIP-1b (75)	FucmAdMSCs 0h replicate 1	15,00	1,00	1,41	9,43	OOR <	*1.41
Mo MIP-1b (75)	FucmAdMSCs 0h replicate 2	14,50	0,50	0,71	4,88	OOR <	*0.98
Mo MIP-1b (75)	FucmAdMSCs 0h replicate 3	14,50	0,50	0,71	4,88	OOR <	*0.98
Mo MIP-1b (75)	FucmAdMSCs 4h replicate 1	34,00	20,00	1,41	4,16	11,84	11,84
Mo MIP-1b (75)	FucmAdMSCs 4h replicate 2	36,50	22,50	0,71	1,94	12,97	12,97
Mo MIP-1b (75)	FucmAdMSCs 4h replicate 3	48,50	34,50	3,54	7,29	18,09	18,09
Mo MIP-1b (75)	FucmAdMSCs 12h replicate 1	86,00	72,00	1,41	1,64	32,22	32,22
Mo MIP-1b (75)	FucmAdMSCs 12h replicate 2	91,00	77,00	4,24	4,66	33,98	33,98
Mo MIP-1b (75)	FucmAdMSCs 12h replicate 3	89,50	75,50	0,71	0,79	33,45	33,45
Mo MIP-1b (75)	FucmAdMSCs 24h replicate 1	41,00	27,00	0,00	0,00	14,95	14,95
Mo MIP-1b (75)	FucmAdMSCs 24h replicate 2	64,50	50,50	2,12	3,29	24,38	24,38
Mo MIP-1b (75)	FucmAdMSCs 24h replicate 3	75,30	61,30	0,35	0,47	28,37	28,37
Mo MIP-1b (75)	FucmAdMSCs 48h replicate 1	56,50	42,50	4,95	8,76	21,29	21,29
Mo MIP-1b (75)	FucmAdMSCs 48h replicate 2	112,50	98,50	4,95	4,40	41,28	41,28
Mo MIP-1b (75)	FucmAdMSCs 48h replicate 3	113,80	99,80	8,84	7,77	41,69	41,69
Mo MIP-1b (75)	FucmAdMSCs 72h replicate 1	89,50	75,50	0,71	0,79	33,45	33,45
Mo MIP-1b (75)	FucmAdMSCs 72h replicate 2	134,00	120,00	2,83	2,11	48,28	48,28
Mo MIP-1b (75)	FucmAdMSCs 72h replicate 3	121,30	107,30	5,30	4,37	44,16	44,16
Mo MIP-1b (75)	R150	58,70	44,70	3,06	5,21	88,56	88,56
Mo MIP-1b (75)	R151	59,00	45,00	0,00	0,00	89,08	89,08
Mo RANTES (55)	Reagent Name: Background0	25,00	25,00	0,00	0,00		
Mo RANTES (55)	UmAdMSCs 0h replicate 1	30,80	5,80	2,47	8,05	OOR <	OOR <
Mo RANTES (55)	UmAdMSCs 0h replicate 2	28,00	3,00	1,41	5,05	OOR <	OOR <
Mo RANTES (55)	UmAdMSCs 0h replicate 3	29,00	4,00	0,00	0,00	OOR <	OOR <
Mo RANTES (55)	UmAdMSCs 4h replicate 1	82,50	57,50	0,71	0,86	6,95	6,95
Mo RANTES (55)	UmAdMSCs 4h replicate 2	141,50	116,50	0,71	0,50	12,08	12,08
Mo RANTES (55)	UmAdMSCs 4h replicate 3	200,50	175,50	7,78	3,88	16,30	16,30
Mo RANTES (55)	UmAdMSCs 12h replicate 1	306,80	281,80	39,95	13,02	22,88	22,88
Mo RANTES (55)	UmAdMSCs 12h replicate 2	579,00	554,00	1,41	0,24	36,97	36,97
Mo RANTES (55)	UmAdMSCs 12h replicate 3	582,30	557,30	45,61	7,83	37,13	37,13
Mo RANTES (55)	UmAdMSCs 24h replicate 1	367,30	342,30	28,64	7,80	26,26	26,26
Mo RANTES (55)	UmAdMSCs 24h replicate 2	307,50	282,50	21,92	7,13	22,92	22,92
Mo RANTES (55)	UmAdMSCs 24h replicate 3	443,00	418,00	11,31	2,55	30,26	30,26
Mo RANTES (55)	UmAdMSCs 48h replicate 1	589,00	564,00	14,14	2,40	37,45	37,45
Mo RANTES (55)	UmAdMSCs 48h replicate 2	436,50	411,50	19,09	4,37	29,93	29,93
Mo RANTES (55)	UmAdMSCs 48h replicate 3	709,80	684,80	15,91	2,24	43,01	43,01
Mo RANTES (55)	UmAdMSCs 72h replicate 1	763,50	738,50	13,44	1,76	45,41	45,41
Mo RANTES (55)	UmAdMSCs 72h replicate 2	963,00	938,00	12,02	1,25	53,97	53,97
Mo RANTES (55)	UmAdMSCs 72h replicate 3	712,00	687,00	25,46	3,58	43,11	43,11
Mo RANTES (55)	FucmAdMSCs 0h replicate 1	28,00	3,00	1,41	5,05	OOR <	OOR <

Mo RANTES (55)	FucmAdMSCs 0h replicate 2	28,80	3,80	1,06	3,69	OOR <	OOR <
Mo RANTES (55)	FucmAdMSCs 0h replicate 3	26,50	1,50	0,71	2,67	OOR <	OOR <
Mo RANTES (55)	FucmAdMSCs 4h replicate 1	204,50	179,50	0,71	0,35	16,57	16,57
Mo RANTES (55)	FucmAdMSCs 4h replicate 2	295,50	270,50	6,36	2,15	22,22	22,22
Mo RANTES (55)	FucmAdMSCs 4h replicate 3	397,80	372,80	22,27	5,60	27,90	27,90
Mo RANTES (55)	FucmAdMSCs 12h replicate 1	842,30	817,30	12,37	1,47	48,85	48,85
Mo RANTES (55)	FucmAdMSCs 12h replicate 2	1085,50	1060,50	40,31	3,71	59,02	59,02
Mo RANTES (55)	FucmAdMSCs 12h replicate 3	992,00	967,00	29,70	2,99	55,18	55,18
Mo RANTES (55)	FucmAdMSCs 24h replicate 1	262,50	237,50	13,44	5,12	20,26	20,26
Mo RANTES (55)	FucmAdMSCs 24h replicate 2	538,30	513,30	29,34	5,45	35,01	35,01
Mo RANTES (55)	FucmAdMSCs 24h replicate 3	1077,80	1052,80	80,96	7,51	58,71	58,71
Mo RANTES (55)	FucmAdMSCs 48h replicate 1	500,00	475,00	28,28	5,66	33,14	33,14
Mo RANTES (55)	FucmAdMSCs 48h replicate 2	984,00	959,00	16,97	1,72	54,85	54,85
Mo RANTES (55)	FucmAdMSCs 48h replicate 3	1180,50	1155,50	97,58	8,27	62,86	62,86
Mo RANTES (55)	FucmAdMSCs 72h replicate 1	672,00	647,00	35,36	5,26	41,30	41,30
Mo RANTES (55)	FucmAdMSCs 72h replicate 2	1316,00	1291,00	1,41	0,11	68,22	68,22
Mo RANTES (55)	FucmAdMSCs 72h replicate 3	1244,30	1219,30	110,66	8,89	65,39	65,39
Mo RANTES (55)	R150	156,50	131,50	11,03	7,05	52,84	52,84
Mo RANTES (55)	R151	191,00	166,00	3,00	1,57	62,64	62,64
Mo TNF-a (21)	Reagent Name: Background0	16,00	16,00	0,00	0,00		
Mo TNF-a (21)	UmAdMSCs 0h replicate 1	19,00	3,00	1,41	7,44	5,84	5,84
Mo TNF-a (21)	UmAdMSCs 0h replicate 2	18,50	2,50	0,71	3,82	5,21	5,21
Mo TNF-a (21)	UmAdMSCs 0h replicate 3	17,50	1,50	0,71	4,04	3,92	3,92
Mo TNF-a (21)	UmAdMSCs 4h replicate 1	21,00	5,00	1,41	6,73	8,34	8,34
Mo TNF-a (21)	UmAdMSCs 4h replicate 2	22,50	6,50	0,71	3,14	10,18	10,18
Mo TNF-a (21)	UmAdMSCs 4h replicate 3	24,50	8,50	2,12	8,66	12,60	12,60
Mo TNF-a (21)	UmAdMSCs 12h replicate 1	23,80	7,80	0,35	1,49	11,69	11,69
Mo TNF-a (21)	UmAdMSCs 12h replicate 2	81,80	65,80	74,60	91,25	76,71	76,71
Mo TNF-a (21)	UmAdMSCs 12h replicate 3	30,80	14,80	0,35	1,15	19,99	19,99
Mo TNF-a (21)	UmAdMSCs 24h replicate 1	27,50	11,50	0,71	2,57	16,18	16,18
Mo TNF-a (21)	UmAdMSCs 24h replicate 2	25,50	9,50	0,71	2,77	13,80	13,80
Mo TNF-a (21)	UmAdMSCs 24h replicate 3	28,50	12,50	2,12	7,44	17,36	17,36
Mo TNF-a (21)	UmAdMSCs 48h replicate 1	33,50	17,50	0,71	2,11	23,19	23,19
Mo TNF-a (21)	UmAdMSCs 48h replicate 2	29,50	13,50	0,71	2,40	18,53	18,53
Mo TNF-a (21)	UmAdMSCs 48h replicate 3	32,00	16,00	0,00	0,00	21,45	21,45
Mo TNF-a (21)	UmAdMSCs 72h replicate 1	41,30	25,30	3,18	7,71	32,07	32,07
Mo TNF-a (21)	UmAdMSCs 72h replicate 2	48,00	32,00	0,00	0,00	39,68	39,68
Mo TNF-a (21)	UmAdMSCs 72h replicate 3	37,50	21,50	3,54	9,43	27,79	27,79
Mo TNF-a (21)	FucmAdMSCs 0h replicate 1	17,00	1,00	0,00	0,00	OOR <	*3.27
Mo TNF-a (21)	FucmAdMSCs 0h replicate 2	18,50	2,50	0,71	3,82	5,21	5,21
Mo TNF-a (21)	FucmAdMSCs 0h replicate 3	18,00	2,00	1,41	7,86	4,57	4,57
Mo TNF-a (21)	FucmAdMSCs 4h replicate 1	25,50	9,50	0,71	2,77	13,80	13,80
Mo TNF-a (21)	FucmAdMSCs 4h replicate 2	23,50	7,50	0,71	3,01	11,39	11,39
Mo TNF-a (21)	FucmAdMSCs 4h replicate 3	28,00	12,00	0,00	0,00	16,77	16,77
Mo TNF-a (21)	FucmAdMSCs 12h replicate 1	30,50	14,50	0,71	2,32	19,70	19,70
Mo TNF-a (21)	FucmAdMSCs 12h replicate 2	33,00	17,00	2,83	8,57	22,61	22,61
Mo TNF-a (21)	FucmAdMSCs 12h replicate 3	33,00	17,00	1,41	4,29	22,61	22,61
Mo TNF-a (21)	FucmAdMSCs 24h replicate 1	23,50	7,50	0,71	3,01	11,39	11,39
Mo TNF-a (21)	FucmAdMSCs 24h replicate 2	26,50	10,50	0,71	2,67	14,99	14,99
Mo TNF-a (21)	FucmAdMSCs 24h replicate 3	29,50	13,50	2,12	7,19	18,53	18,53
Mo TNF-a (21)	FucmAdMSCs 48h replicate 1	26,00	10,00	1,41	5,44	14,39	14,39
Mo TNF-a (21)	FucmAdMSCs 48h replicate 2	36,50	20,50	0,71	1,94	26,65	26,65
Mo TNF-a (21)	FucmAdMSCs 48h replicate 3	36,00	20,00	2,83	7,86	26,07	26,07
Mo TNF-a (21)	FucmAdMSCs 72h replicate 1	34,00	18,00	1,41	4,16	23,77	23,77
Mo TNF-a (21)	FucmAdMSCs 72h replicate 2	41,50	25,50	2,12	5,11	32,35	32,35
Mo TNF-a (21)	FucmAdMSCs 72h replicate 3	41,00	25,00	2,83	6,90	31,78	31,78
Mo TNF-a (21)	R150	146,80	130,80	8,81	6,00	583,04	583,04
Mo TNF-a (21)	R151	153,30	137,30	12,66	8,26	610,23	610,23

Supplementary Table 1. Secretome raw data used to determine the concentration of each analyte produced in conditioned media by UmAdMSCs or FucmAdMSCs at different times of culture by magnetic bead-based assays in a Luminex system. Beads count detected for each anaylyte are indicated in parentheses. Fl: Fluorescence; Fl-Bkgd: Fluorescence background; Std Dev: Standard deviation; %CV: Percentage of coefficient of variation (inter-assay precision); Conc in Range: Concentration in range; Obs Conc: Observed concentration.
