# Supplementary material for: Exofucosylation of Adipose Mesenchymal Stromal Cells Alters Their Secretome Profile
Source: Front Cell Dev Biol. 2020 Nov 26;8:584074. doi: 10.3389/fcell.2020.584074 (PMC7726227; doi:10.3389/fcell.2020.584074)
Supplement: Supplementary file 4 [file Data_Sheet_4.PDF]

| Gene name | Gene | Sequence 5´- 3´ | Accesion number |
|---|---|---|---|
| Transforming growth factor $\beta$ | Tgfb | Fwd: GGATACCAACTATTGCTTCAG | NM_011577 |
| | | Rev: TGTCCAGGCTCCAAATATAG | |
| Interleukin-10 | Il10 | Fwd: CAGGACTTTAAGGGTTACTTG | NM_010548 |
| | | Rev: ATTTTCACAGGGGAGAAATC | |
| Indoleamine 2,3-dioxygenase 1 | Ido1 | Fwd: TTTTTAAGAGGATGCGTGAC | NM_008324.2 |
| | | Rev: TATTCCCAGAAGGACATCAAG | |
| Hepatocyte growth factor | Hgf | Fwd: CAAATGCAAGGACCTTAGAG | NM_010427 |
| | | Rev: CTTGTTTTGGATAAGTTGCC | |
| Heme oxygenase-1 | Hmox1 | Fwd: CATGAAGAACTTTCAGAAGGG | NM_010442 |
| | | Rev: TAGATATGGTACAAGGAAGCC | |
| Galectin-1 | Lgals1 | Fwd: CAGGTCTCAGGAATCTCTTC | NM_008495 |
| | | Rev: CAGGTTTGAGATTCAGGTTG | |
| Cyclooxygenase-1 | Ptgs1 | Fwd: CATCCTTATAGGAGAAACCATC | NM_008969 |
| | | Rev: GTTTCGATATTGGAACTGGG | |
| Cyclooxygenase-2 | Ptgs2 | Fwd: ACTCATAGGAGAGACTATCAAG | NM_011198 |
| | | Rev: GAGTGTGTTGAATTCAGAGG | |
| Adiponectin | Adipoq | Fwd: ACTCATAGGAGAGACTATCAAG | NM_009605 |
| | | Rev: GAGTGTGTTGAATTCAGAGG | |

| | | | |
|---|---|---|---|
| PPAR$\gamma$2 | Pparg2 | Fwd: ACTCATAGGAGAGACTATCAAG | NM_001127330 |
| | | Rev: GAGTGTGTTGAATTCAGAGG | |
| Alkaline phosphatase | Alp1 | Fwd: ACTCATAGGAGAGACTATCAAG | NM_007431 |
| | | Rev: GAGTGTGTTGAATTCAGAGG | |
| Osteocalcin | Bglap | Fwd: ACTCATAGGAGAGACTATCAAG | NM_001032298 |
| | | Rev: GAGTGTGTTGAATTCAGAGG | |
| Collagen type I, $\alpha$1 chain | Col1a1 | Fwd: ACTCATAGGAGAGACTATCAAG | NM_007742 |
| | | Rev: GAGTGTGTTGAATTCAGAGG | |
| SOX9 | Sox9 | Fwd: CTCATTACCATTTTGAGGGG | NM_011448 |
| | | Rev: AAAATACTCTGGTTGCAAGG | |
| Epiphycan | Epyc | Fwd: CAATAGACTGGGAAGAAAAGG | NM_007884 |
| | | Rev: CATTATTCTGGAGGTGAAGAG | |
| Glyceraldehyde-3-phosphate dehydrogenase | Gapdh | Fwd: AGGTCGGTGTGAACGGATTTG | NM_008084 |
| | | Rev: GGGGTCGTTGATGGCAACA | |

**Supplementary Table 2.** List of primer sequences used in this study for qPCR analysis. Fwd: Forward primer. Rev: Reverse primer.
