# Supplementary material for: Exofucosylation of Adipose Mesenchymal Stromal Cells Alters Their Secretome Profile
Source: Front Cell Dev Biol. 2020 Nov 26;8:584074. doi: 10.3389/fcell.2020.584074 (PMC7726227; doi:10.3389/fcell.2020.584074)
Supplement: Supplementary file 5 [file Data_Sheet_5.PDF]

Surface marker	UmAdMSCs				FucmAdMSCs			*p* %
	0h	24h	48h	72h	24h	48h	72h	
**CD73**	96.06 ± 2.04	96.77 ± 1.38	96.88 ± 2.02	97.80 ± 1.39	96.52 ± 1.61	97.77 ± 1.28	97.73 ± 0.64	0.810
**CD105**	99.06 ± 0.79	98.21 ± 0.16	98.06 ± 0.71	98.81 ± 0.85	97.98 ± 0.18	98.78 ± 0.76	98.99 ± 0.03	0.647
**CD90**	97.32 ± 1.13	96.82 ± 1.30	98.44 ± 1.22	98.90 ± 0.53	97.23 ± 1.75	98.28 ± 1.06	98.49 ± 1.02	0.948
**CD44**	99.65 ± 0.31	99.21 ± 0.62	99.31 ± 0.70	99.59 ± 0.20	99.36 ± 0.69	98.48 ± 0.22	98.78 ± 0.64	0.239
**CD29**	99.51 ± 0.29	99.59 ± 0.29	99.43 ± 0.30	99.31 ± 0.39	99.47 ± 0.35	99.85 ± 0.09	99.33 ± 0.33	0.553
**CD166**	16.75 ± 2.26	19.92 ± 1.64	18.80 ± 1.32	19.81 ± 2.39	19.17 ± 3.93	19.73 ± 2.58	20.02 ± 1.87	0.929
**CD106**	88.58 ± 2.23	81.72 ± 1.81	82.72 ± 4.67	86.69 ± 6.11	81.97 ± 3.01	83.03 ± 3.65	85.02 ± 2.47	0.902
**Sca-1**	99.47 ± 0.37	99.57 ± 0.14	99.51 ± 0.08	99.26 ± 0.44	99.03 ± 0.28	99.44 ± 0.37	99.34 ± 0.30	0.325

Surface marker	UmAdMSCs				FucmAdMSCs			*p* MFI
	0h	24h	48h	72h	24h	48h	72h	
**CD73**	8.08 ± 1.91	8.93 ± 0.79	9.31 ± 1.35	9.67 ± 1.49	9.10 ± 1.30	9.72 ± 0.93	10.12 ± 2.07	0.665
**CD105**	16.67 ± 0.70	15.56 ± 0.69	16.14 ± 1.72	17.37 ± 0.74	16.66 ± 1.12	16.07 ± 1.66	17.21 ± 1.09	0.641
**CD90**	6.65 ± 0.83	7.25 ± 0.57	7.49 ± 0.81	8.26 ± 0.26	7.47 ± 0.53	7.69 ± 0.42	7.99 ± 0.76	0.929
**CD44**	23.71 ± 2.17	27.75 ± 1.43	25.66 ± 1.33	25.77 ± 0.69	26.11 ± 2.11	25.32 ± 2.71	25.59 ± 1.76	0.598
**CD29**	12.11 ± 0.42	13.76 ± 0.96	11.97 ± 1.49	12.07 ± 0.95	14.40 ± 1.24	11.26 ± 0.99	12.71 ± 1.31	0.864
**CD166**	3.21 ± 1.86	6.36 ± 1.14	4.92 ± 0.34	5.09 ± 0.78	6.14 ± 1.44	5.32 ± 1.02	5.53 ± 0.57	0.869
**CD106**	9.15 ± 1.06	8.59 ± 0.46	8.39 ± 0.51	8.46 ± 0.77	8.31 ± 0.26	8.20 ± 0.91	8.53 ± 0.43	0.724
**Sca-1**	15.97 ± 1.31	18.02 ± 0.90	18.01 ± 1.15	20.70 ± 0.94	18.22 ± 1.22	19.07 ± 0.68	20.09 ± 1.21	0.904

Supplementary Table 3. Evolution of expression of positive MSC markers on mAdMSCs after *ex vivo* fucosylation. UmAdMSCs or FucmAdMSCs was cultured for the indicated times and analyzed for expression of the typical mesenchymal stem cell markers CD73, CD105, CD90, CD44, CD29, CD166, CD106 and Sca-1 by flow cytometry. Data represent the mean ± SD of percentage of positive cells (top panel) or the mean fluorescence intensity values (MFI) (bottom panel) for each surface marker from three independent experiments. $p>0.05$, using one-way ANOVA and Tukey's post-hoc comparison tests.
